# Evaluating the Molecular Basis of Nanocalcium-Induced Health Regulation in Zebra Fish (*Danio rerio*)

**DOI:** 10.3390/bioengineering12101016

**Published:** 2025-09-24

**Authors:** Madhubala Kumari, Aastha Tiwary, Rishav Sheel, Arnab Roy Chowdhury, Biplab Sarkar, Koel Mukherjee, Dipak Maity

**Affiliations:** 1 Department of Bioengineering and Biotechnology, Birla Institute of Technology, Mesra, Ranchi 835215, Jharkhand, India; 2 School of Molecular Diagnostics, Prophylactics and Nanobiotechnology, ICAR-Indian Institute of Agricultural Biotechnology (IIAB), Garhkhatanga, Ranchi 834003, Jharkhand, India; 3 Downstream Agro-Processing Division, ICAR-National Institute of Secondary Agriculture (NISA), Namkum, Ranchi 834010, Jharkhand, India; 4 Integrated Nanosystem Development Institute, Indiana University Indianapolis, Indianapolis, IN 46202, USA; 5 Department of Chemistry and Chemical Biology, Indiana University Indianapolis, Indianapolis, IN 46202, USA

**Keywords:** calcium oxide nanoparticles, *Danio rerio*, histological analysis, molecular docking, NRF2 pathway, aquaculture

## Abstract

The present study aimed to evaluate the impact of varying dietary concentrations of calcium oxide nanoparticles (CaO-NPs) on important health regulators in Zebra fish (*Danio rerio)* using integrative physiological, histopathological, and computational approaches. The co-precipitation method was used to synthesize NPs and characterization was performed through DLS, XRD, FESEM, EDX, and FTIR depicting spherical-shaped CaO-NPs with a hydrodynamic diameter of 91.2 nm. Adult *Danio rerio* were administered with three different feed regimes enriched with 2.4 (T1), 1.6 (T2), and 0.8 (T3) mg CaO-NPs/kg for 30 days. Growth, survival, NP accumulation, and histological assessments, and bioinformatic studies, were performed to understand interactions of NPs with fish metabolic proteins. The T3 group demonstrated the highest survival (75%) and weight gain (+39.31%), and exhibited the lowest accumulation of CaO-NPs in the brain (0.133 mg/L), liver (0.642 mg/L), and intestine (0.773 mg/L) with no evident histological alterations, whereas T1 group exhibited major liver and intestinal damage. Molecular docking targeting the NRF-2 oxidative stress pathway revealed strong binding affinities of NPs with catalase (−3.7), keap1a (−3.5), keap1b (−3.3), and mafk (−2.4), highlighting potential modulation of redox homeostasis. Hence, a 0.8mg CaO-NPs/kg feed dose is recommended to promote potential health benefits in *Danio rerio*, which can be further applicable to commercial aquaculture for enhanced fish health while minimizing toxicity.

## 1. Introduction

The aqua-feed industry experienced significant growth over the past decade, contributing approximately 52% of the global fish supply in 2020 compared to 40% in 2010 [1]. This growth reflects increasing reliance on aquaculture to meet the rising global demand for sustainable protein sources. The sector is projected to grow at a compound annual growth rate (CAGR) of 5.8% by 2030, reaching a global market value of USD 285 billion. As the industry continues to expand, sustainable innovations in feed formulations are essential to enhance growth performance and fish health while minimizing ecological impact [2].

Minerals such as calcium are integral to fish physiology, contributing to bone development, muscle contraction, nerve function, blood clotting, and maintaining cellular membrane stability [3,4]. Adequate calcium levels also stabilize gill function and osmoregulation, mitigating stress responses in varying environmental conditions. Calcium ions (Ca^2+^) primarily absorbed through gills and the gastrointestinal tract, yet traditional supplementation strategies often suffer from poor bioavailability, leading to inefficient uptake and potential deficiencies [5,6].

Current advances in nanotechnology offer novel delivery strategies, notably CaO-NPs, which may enhance absorption and physiological function due to their versatility and potential benefits [7]. CaO-NPs are emerging as a promising alternative due to their high surface area, small size, and enhanced reactivity, which collectively facilitate better absorption and targeted delivery [7]. While these properties suggest potential health benefits, concerns persist regarding the bioaccumulation of CaO-NPs in vital organs and their possible toxicity at elevated doses [8,9]. Previous studies have largely focused on acute toxicity and mortality, with limited understanding of sub-lethal or chronic effects on organ function, histology, and molecular pathways related to oxidative stress [10,11,12].

A key pathway implicated in cellular defence against nanoparticle-induced toxicity is the NRF2 (Nuclear Factor Erythroid 2–Related Factor 2) signalling cascade. NRF2 is a redox-sensitive transcription factor that governs the expression of various antioxidant and cytoprotective genes. Under normal conditions, NRF2 is sequestered in the cytoplasm by its repressor KEAP1. Upon oxidative challenge, such as exposure to reactive nanoparticles, NRF2 dissociates, translocates to the nucleus, and activates antioxidant response elements (AREs), triggering a cellular defence response [13,14,15]. Disruption or modulation of this pathway by nanoparticles can significantly alter redox balance, immune response, and cellular homeostasis in aquatic organisms [16,17,18]. However, the interaction of CaO-NPs with cellular redox mechanisms, particularly the NRF2-regulated antioxidant pathway remains poorly characterized in aquatic species. Understanding the biological interactions of nanoparticles, including their accumulation in tissues and potential to induce cellular or molecular changes, is crucial [8,9]. Key areas of investigation include the impact of nanoparticles on fish growth, tissue structure, and molecular pathways, as well as their potential to cause oxidative stress or other adverse effects [10]. Factors such as nanoparticle size and concentration influence their interaction with biological tissues, underscoring the need for comprehensive assessments of their implications for sustainability in aquaculture and animal health [11,12,13].

Zebra fish (*Danio rerio*) has emerged as a valuable model organism in aquatic toxicology and nutritional research due to its genetic homology with mammals, transparent body, and rapid development cycle. Its well-characterized genome and amenability to molecular assays make it ideal for evaluating physiological and toxicological impacts of engineered nanomaterials [14,19]. Importantly, *D. rerio* has proven useful in elucidating oxidative stress pathways, including NRF2 signalling, in response to environmental toxins and dietary interventions [14].

Given the increasing interest in applying CaO-NPs to aqua-feed, it is critical to assess both the beneficial and adverse effects of these materials at a molecular and organismal level. This study was designed to evaluate the dose-dependent effects of CaO-NP-enriched diets on *D. rerio* health using a combined in vivo and in silico approach. By analyzing metabolic pathways and regulatory protein networks within *D. rerio*, hub genes identification and the binding efficiency of CaO-NPs to these proteins using molecular docking tools were accessed. The study hypothesizes that CaO-NPs strongly bind to proteins involved in critical physiological functions, potentially disrupting processes such as oxidative stress management, cellular defence, and development. This interaction, particularly in the liver and brain may lead to oxidative stress, apoptosis, and cellular dysfunction, affecting normal organ function [16,17,18].

Despite increased interest in CaO-NPs for aquaculture applications, numerous crucial areas remain unexplored. The majority of previous studies were focused on NPs’ acute toxicity, while the long-term consequences of chronic sub-lethal exposure on fish physiology and growth status are poorly understood [14,19]. Besides these aspects, more research initiatives are needed into the molecular mechanisms induced by CaO-NPs, especially their interactions with cellular components. Bridging these gaps is critical for ensuring the safe and sustainable use of CaO-NPs in aquaculture.

Hence, the present study was designed to address key knowledge gaps concerning the application of CaO-NPs as a feed additive for fishes. A combination of in vivo and in silico approaches was employed to comprehensively evaluate their effects. The in vivo experiments focused on assessing the dose-dependent impacts of CaO-NP-enriched fish feed on growth performance, histopathological changes, and organ-specific nanoparticle accumulation in *D. rerio*. In parallel, nano-informatics tools were utilized to investigate the molecular interactions between CaO-NPs and NRF-2, a pivotal transcription factor involved in oxidative stress regulation, along with other key metabolic proteins. This integrated approach aimed to elucidate the physiological and molecular responses associated with CaO-NP exposure and to determine their safety and functional potential in aqua-feed formulations by identifying safe dosage ranges that maximize growth and health benefits while minimizing potential toxicological risks.

## 2. Experimental Methods

### 2.1. CaO-NP Synthesis

CaO-NPs were chemically synthesized using a co-precipitation method [20]. A 0.03 M calcium nitrate (CaNO_3_; Himedia; Bhiwandi, Thane, Mumbai) solution was prepared in distilled water and 0.06 M sodium hydroxide (NaOH; Himedia; Bhiwandi, Thane, Mumbai) solution was added drop wise with continuous stirring for complete mixing till the development of a white precipitate at the bottom of the glassware. Then the solution was further continuously stirred (Remi, Vasai, India) for 1.5 h and kept overnight (27–29 °C) before being filtered. Thereafter, the solution was centrifuged (Remi, Vasai, India) at 12,000 rpm for 10–15 min and washed simultaneously to remove excess alkalinity. Then the white pellets were collected and kept for proper drying through calcination at 400 °C for 4h in a muffle furnace at the end of the procedure. The resulting yield of CaO-NPs was ground to a fine powder using a mortar and pestle. Then the CaO-NPs were dispersed in Milli-Q water and probe-sonicated for 10–15 min at 2 pulses and 25 amplitudes (A), and further characterized using various techniques.

### 2.2. Physiochemical Characterization of CaO-NPs

After the synthesis of CaO-NPs, characterizations were performed by applying various high-throughput instruments. The details of all the performed techniques are given below.

#### 2.2.1. Dynamic Light Scattering (DLS)

The CaO-NP size and zeta potential measurements were performed with a particle size analyser (Anton Paar Litesizer 500, Anton Paar GmbH, Graz, Austria). The parameters used were He-Ne, 4.0 mW, 633 nm laser, and a 173 degree measurement angle. This technique was conducted on a suspended CaO-NP sample, where the NPs displayed Brownian motion in the solvent. This method relies on the principle of light scattering, utilizing the scattered light from CaO-NPs to compute their average size through the polydispersity index (PDI).

#### 2.2.2. X-Ray Diffraction (XRD) Analysis

The phase identification and average crystallite size were measured by a powder X-ray diffractometer (Rigaku Japan; Smart lab 9 KW, Tokyo, Japan) with a 3–120 degree, 2 theta angle range, 3 KW and 9 KW power, and with the help of hypix 3000.

#### 2.2.3. Field Emission Scanning Electron Microscopy (FESEM) and Energy-Dispersive Spectroscopy (EDS) Analysis

Field emission scanning electron microscopy (Sigma 300, Carl Zeiss Germany, Oberkochen, Germany) was employed for the morphological examination and size measurement of CaO-NPs at various scales and magnifications for surface-level investigation.

#### 2.2.4. Fourier Transform Infrared Spectroscopy (FTIR) Analysis

Fourier transform infrared spectroscopy (Perkin Elmer USA; Shelton, Connecticut, Frontier) was used to identify the functional groups present in the CaO-NPs. Here, the detectors used were DTGS and FRDTGSS with a wavelength precision of ±0.1 cm^−1^ with the help of gold-coated mirror optics. A small quantity of CaO-NP powder was added to KBr to form KBr pellets, and then processed for functional group analysis. The absorption of light at specific wavelengths by the CaO-NP samples was assessed using FTIR spectroscopy in the range of 400–4000 cm^−1^ at a resolution of 4 cm^−1^ to assess the probable functional groups related to synthetic approaches.

#### 2.2.5. AFM

AFM analysis (NT-MDT Russia; Solver Pro 47, Zelenograd, Moscow, Russia) was conducted to examine the surface topography of CaO-NPs. The dried NP powder was compressed into small pellets using a pelletizer. These pellets were directly mounted onto the AFM stage, and scanning was carried out in contact mode using a silicon cantilever. Topographical images were recorded over selected scan areas, and surface roughness was analyzed using integrated Nova software Version- 18857.

### 2.3. Physiochemical Characterization of Water Sample

Major water quality parameters like ammonia, nitrate, conductivity, pH, temperature, etc., were measured regularly during starting as well as at intervals of every 15 days during the experimental period using various assays prescribed by the American Public Health Organization [21] (Table S1 in the Supplementary File).

### 2.4. Preparation of CaO-NP-Enriched Fish Diet

An artificial fishmeal-based pellet diet was made by adhering to the methodology described earlier [22]. The diet formulation included careful measured quantities of dry ingredients and vitamin mixes, as specified in Table S2 (Supplementary File). These components were thoroughly blended using a laboratory-grade food mixer (Bajaj 3701 GX 750 W mixer grinder) to ensure uniformity. To investigate the effects of nanocalcium supplementation, chemically synthesized CaO-NPs were incorporated into the diet at three distinct concentrations: 2.4 (T1), 1.6 (T2), and 0.8 (T3) mg CaO-NPs/kg of feed. A positive control diet was also prepared, containing calcium nitrate at a concentration of 2.4 mg/kg of feed, along with a negative control diet, including all ingredients except any additional calcium source. The blended ingredients were mixed with distilled water to create uniform dough, which was then subjected to autoclaving for sterilization. Following sterilization, the dough was processed into 500 µm pellets using a pelletizer. These pellets were air-dried for 6–7 h to achieve complete dryness and then stored in air-tight containers for subsequent feeding experiments.

### 2.5. Acclimatization of D. rerio

Adult *D. rerio* (30 days post-hatching) were procured from a certified aquaculture supplier in Ranchi, Jharkhand, India, to ensure a reliable and healthy stock for experimentation. Upon arrival, the fish were subjected to a prophylactic treatment with a 0.05% potassium permanganate (KMnO_4_) solution. This immersion treatment was performed twice, with each session lasting 1–2 min and an adequate recovery interval between treatments, to effectively reduce the risk of dermal and ectoparasitic infections. Following the prophylactic treatment, the fish were carefully transferred to aerated experimental tubs and allowed to acclimatize to laboratory conditions for a period of two weeks. During this acclimatization phase, water quality parameters, including temperature (26 ± 1 °C), pH (7.0–7.5), dissolved oxygen (≥6 mg/L), and ammonia levels (≤0.02 mg/L), were meticulously maintained to simulate optimal aquatic conditions.

### 2.6. Feeding Trial and Statistical Analysis on Growth Performance of D. rerio

After proper acclimatization, 25 healthy fishes were randomly added to a polyfibre tub with a 30L water holding capacity with five experimental groups (2.4, 1.6, and 0.8 mg CaO-NPs/Kg feed, as well as negative and positive controls, denoted as T1, T2, T3, NC, and PC). Prior to the experiment, the average length and weight of the fish were recorded as 4.15 ± 0.2 cm and 0.42 ± 0.1 g, respectively. Triplicates were maintained for each feeding regime, with a 12 h light/12 h dark photoperiod. The fish were fed twice daily at 2% of their body weight and uneaten feed was removed to prevent water contamination [23]. Average body weight was measured on the 1st and at 30th day of the experimental period to monitor general growth performance. A survival chart was maintained throughout the experiment, and deceased fish were promptly removed to prevent infection and water contamination. The weight gain % in fishes in each group was measured using the formula given below:

Weight gain % = (Final weight − Initial weight)/Initial weight × 100

Additionally, one-way analysis of variance (ANOVA) was performed using OriginPro 8.5 to assess the effect of different concentrations of CaO-NPs on *D. rerio* growth parameters. Data were tested for normality using the Shapiro–Wilk test, and homogeneity of variances was confirmed using Levene’s test. One-way ANOVA was applied to compare the mean weight among different groups, and Tukey’s post hoc test was conducted for pairwise comparisons. A *p*-value < 0.05 was considered statistically significant [24]. The mean and standard deviation for each group were provided, which gave an overview of the central tendency and variability in each group.

### 2.7. Tissue Sample Collection

At the end of the 30-day experimental period, all fishes were anesthetized using 0.04% clove oil to ensure minimal stress and pain. After rinsing the fish in dechlorinated water to remove residual anesthetic, dissection was performed on a clean, sterile surface using sterilized tools. Organs such as the liver, intestine, and brain were identified, removed, and rinsed with phosphate-buffered saline (PBS) to eliminate blood and contaminants. Collected organs from individual fish were pooled into pre-labelled sterile tubes and processed for storage based on the requirements of downstream analyses: tissues for histology were fixed in 10% formalin, and elemental analysis samples were stored at −80 °C. After dissection, carcasses were disposed of following the institutional regulations [25].

### 2.8. Histological Test

After 30 days of exposure, *D. rerio* specimens from each experimental group were dissected and fixed in a 10% formalin solution for histopathological examination following a previously prescribed method [26]. The tissues were then rinsed with running tap water and dehydrated using a graded series of ethanol. Following dehydration, the tissues were cleaned in xylene and embedded in paraffin blocks. Sections of the tissues were cut at a thickness of 5 μm using a microtome (Medimeas, Ambala Cantt., India). These sections were mounted on clean slides and stained with hematoxylin and eosin (SRL, Chakala, Andheri East, India). Histopathological changes were then observed using a light microscope (Leica, Wetzlar, Germany) [27]. This meticulous process ensures accurate and detailed histopathological analysis, providing crucial insights into the tissue alterations induced by the experimental conditions.

### 2.9. CaO-NP Accumulation Analysis Using ICP-OES

Microwave digestion of the tissue samples was carried out to prepare them for elemental analysis. After 30 days of exposure, 0.2 g of each *D. rerio* tissue (liver, intestine, and brain) was precisely weighed and placed into closed polytetrafluoroethylene (PTFE) digestion vessels. To ensure efficient digestion, the tissues were mixed with a reagent solution comprising nitric acid (HNO_3_) and hydrogen peroxide (H_2_O_2_) in an 8:2 volume ratio. This acidic mixture aids in breaking down the organic matrix of the tissue while ensuring complete mineralization of the samples [28]. The sealed vessels were subjected to control microwave (Anton Paar Multiwave 3000, Graz, Austria) heating for a duration of two hours, during which the elevated temperature and pressure facilitated the digestion process, yielding a clear solution suitable for further analysis. After digestion, the resulting solutions were carefully transferred to pre-labelled volumetric flasks. Milli-Q water, high-purity deionized water, was added to each flask to dilute the samples to a final volume of 100 mL. The diluted solutions were then passed through Whatman grade 42 filter paper, ensuring clarity of the samples. The prepared samples were subsequently analyzed for calcium accumulation across various tissue types and experimental groups using the Inductively Coupled Plasma Optical Emission Spectroscopy (ICP-OES) technique (2100D; Perkin Elmer, Shelton, USA). The quantification of calcium was achieved using a calibration curve created with known standards, ensuring accuracy and reliability in the measurement.

### 2.10. Computational Methods

#### 2.10.1. Selection of Metabolic Pathway, Retrieval of Proteins and Its 3D Structure

To enhance analytical precision, two parameters were selected for protein analysis: the NRF2 pathway and organ-specific proteins. The functions of all the selected proteins are mentioned in Table S3 (Supplementary File). Nuclear factor erythroid 2-related factor 2 (NRF2) is a key regulator in calcium signalling, cytotoxicity, and cellular defence against oxidative stress, particularly induced by CaO-NPs. Given its importance, the NRF2 pathway was chosen for a detailed molecular-level investigation of oxidative stress and nanotoxicity caused by CaO-NPs in adult *D. rerio*. For functional analysis of protein pathways, the KEGG (Kyoto Encyclopedia of Genes and Genomes) database (https://www.genome.jp/kegg/pathway.html; accessed on 24 October 2024) was used, providing graphical representations of cellular processes, such as metabolism, signal transduction, and the cell cycle, which enabled a comprehensive view of gene functions and biological systems [29]. Additionally, a focused study on the calcium metabolism pathway was conducted in *D. rerio*, identifying key proteins involved in metabolic pathways and essential for growth and development in four targeted organs: liver, intestine, brain, and gills. Protein sequences for these analyses were obtained from the NCBI database in FASTA format (https://www.ncbi.nlm.nih.gov/; accessed on 24 October 2024) [30]. Furthermore, protein–protein interactions among the proteins involved in the NRF2 pathway were analyzed using the STRING database (Version- 12.0), employing a full STRING network with a medium confidence score (0.400) and a size cut-off of ≤20 interactors to enhance the accuracy of the analysis [31].

#### 2.10.2. Retrieval of CaO-NP 3D Structure

The three-dimensional crystal structure of CaO-NPs was generated from the Materials Project database (https://next-gen.materialsproject.org/; accessed on 29 October 2024). The CaO-NPs exhibited a cubic crystal structure (mp-2605), classified under the space group symbol Fm3m, with a band gap of 3.63 eV. The Crystallographic Information File (CIF) produced by this database was subsequently converted into the Protein Data Bank (PDB) format using PyMOL software (version-3.0), facilitating compatibility for docking studies.

#### 2.10.3. Three-Dimensional Modelling and Verification

The three-dimensional structures of some of the selected proteins were not available in the Protein Data Bank (PDB). Therefore, these structures were modelled using MODELLER software version 10.4 (https://salilab.org/modeller/download_installation.html; Accessed on 30 October 2024) [32]. The quality of the best-modelled structures was then assessed using PDBsum software, (http://www.ebi.ac.uk/thornton-srv/databases/pdbsum/Generate.html; Accessed on 30 October 2024) [33]. It provided a Ramachandran plot that indicated the percentage of residues in favoured and disallowed regions, helping to verify the accuracy of the model [34,35] (Table S4 in the Supplementary File).

#### 2.10.4. Molecular Dynamics Study

The structures of proteins obtained by homology modelling were subjected to a 20 ns molecular dynamics simulation using GROMACS (version 2023.2) to assess their stability [36]. The unstructured region of the N and C terminals of the proteins were removed as they hinder the convergence of the simulation and do not provide meaningful insights into the action of the protein. System preparation was accomplished using the solution builder module within CHARMM-GUI. The solution box was set to cubic type (varying from 9 to 12 Å) and the system was solvated using 0.15 M sodium chloride ions and TIP3P water molecules. The system was minimized using the steepest descent integrator. The energy tolerance for convergence was set to 1000.0 kJ/mol with 5000 steps. The neighbour list was updated every 10 steps using the Verlet cut-off scheme with a cut-off radius of 1.2 nm. Additionally, a force-switch modifier is applied for van der Waals interactions, with a switching radius of 1.0 nm and a cut-off radius of 1.2 nm. Constraints were applied to hydrogen bonds using the LINCS algorithm. The system was maintained at 300 K using the Nose–Hoover thermostat and temperature coupling was applied to the solute, i.e., protein. The Parrinello–Rahman barostat was used to maintain the pressure at 1 bar. The system was equilibrated for 250 ps in both NVT and NPT, before the 20 ns unrestrained production runs.

#### 2.10.5. Active Site Prediction, Molecular Docking Analysis of Proteins

The active sites of the selected and modelled proteins were predicted using the CASTp server and the ScanProsite server. The CASTp server (https://www.bing.com/search?q=castp+server&form=ANNTH1&refig=68d232062f5843a2b3e64cdaa28492f4&pc=U531#; accessed on 30 October 2024) identifies surface pockets and interior cavities of proteins, while the ScanProsite server (https://prosite.expasy.org/scanprosite/; accessed on 30 October 2024) detects potential functional motifs.

For the docking studies, the protein and CaO-NPs were docked using the CBDock server (http://clab.labshare.cn/cb-dock/; accessed on 30 October 2024). This protein–ligand docking software utilizes a local shape feature matching algorithm to form complexes. The complex with the highest score indicates the maximum shape complementarities between the protein and ligand molecule. Both protein and ligand structures were uploaded in PDB format and further converted to PDBQT format for the docking process. Post-docking, the resulting complexes were analyzed using PyMOL 3.0 (https://www.pymol.org/; accessed on 30 October 2024) and LigPlot software- Ligplot+ v2.3 (https://www.ebi.ac.uk/thornton-srv/software/LigPlus/; accessed on 30 October 2024). These tools helped visualize the positions or poses of the CaO-NPs within the complex and allowed for the analysis of hydrogen bonds and other interactions formed between the complexes [37]. This comprehensive approach provides insights into the molecular interactions and binding affinities between the proteins and CaO-NPs, which are crucial for understanding their functional implications.

## 3. Results

### 3.1. CaO-NP Synthesis and Characterization

**Figure 1:**
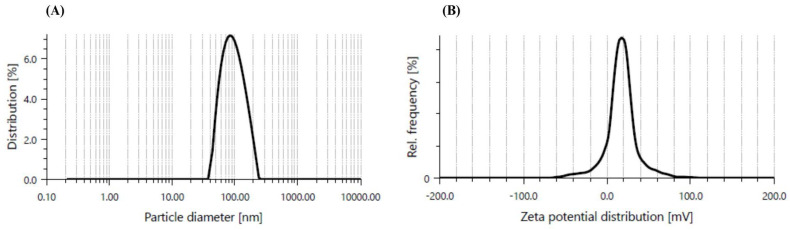
Graphical representation of (**A**) particle size diameter and (**B**) zeta potential distribution of calcium oxide nanoparticles using Dynamic Light Scattering and electrophoretic light scattering, respectively.

CaO-NPs were chemically synthesized and fine white precipitate formation confirmed their synthesis. To further characterize the NPs, several types of characterization techniques were performed. Figure 1 illustrates the particle size distribution and dispersity of the CaO-NPs through Dynamic Light Scattering (DLS). The hydrodynamic diameter obtained by DLS was 91.2 nm with a PDI of 0.215, indicating a narrow size distribution and good dispersibility in aqueous suspension, where the particles had a narrow size distribution, crucial for applications requiring consistency in particle behaviour. Additionally, the zeta potential, measured separately by electrophoretic light scattering (ELS), was +12.0 ± 0.9 mV, which corresponds to moderate electrostatic stability for CaO-NPs in suspension.

**Figure 2:**
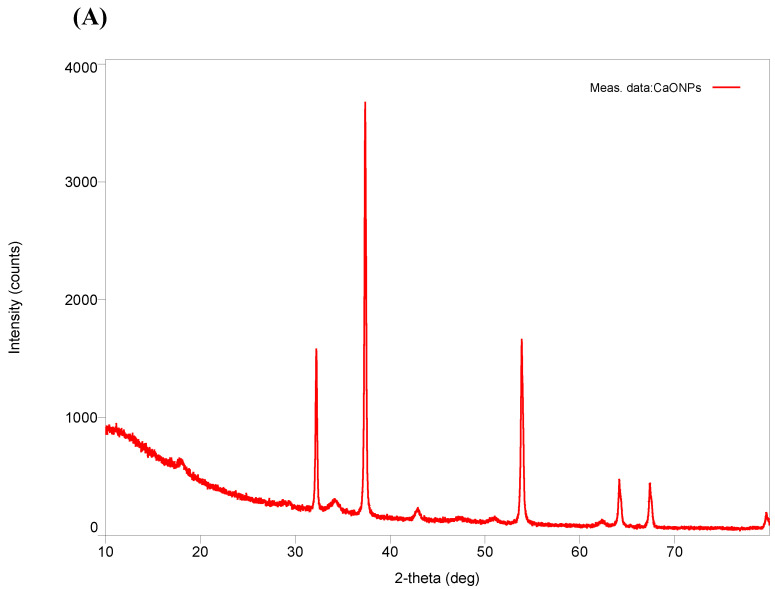

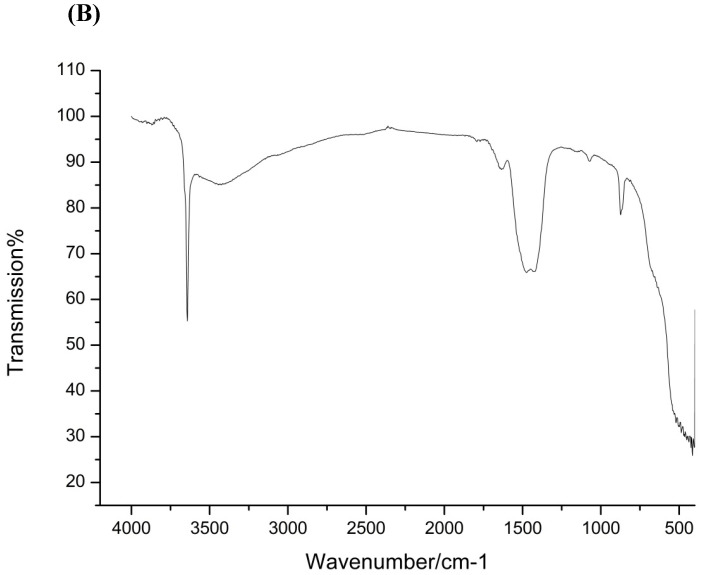
Diagrammatic representation of the characterization of calcium oxide nanoparticles using (**A**) XRD and (**B**) FTIR.

The crystalline structure and functional group analyses were performed using X-ray Diffraction (XRD) and Fourier transform infrared spectroscopy (FTIR) techniques as depicted in Figure 2. X-ray Diffraction (XRD) patterns showed distinct peaks at 2θ values of 32°, 37°, 54°, and 67°, corresponding to the cubic crystalline structure of CaO (JCPDS No. 37-1497). The crystallite size, estimated using the Scherrer equation, ranged from 6.6 nm to 47.8 nm, with the most intense peak at 32.23°. The absence of amorphous peaks indicated the high crystallinity and purity of the synthesized CaO-NPs. The FTIR spectrum showed a peak at 3645 cm^−1^, representing O–H stretching (surface hydroxylation), and a band at 1631 cm^−1^ indicating bending vibrations of adsorbed water molecules. Carbonate stretching vibrations appeared at 1456 cm^−1^, likely due to partial surface carbonation from atmospheric CO_2_.

A band at 1082 cm^−1^ (C–O stretching) supported this, while peaks at 872 cm^−1^ and 618 cm^−1^ were attributed to Ca–O bond vibrations, confirming CaO-NP formation.

**Figure 3:**
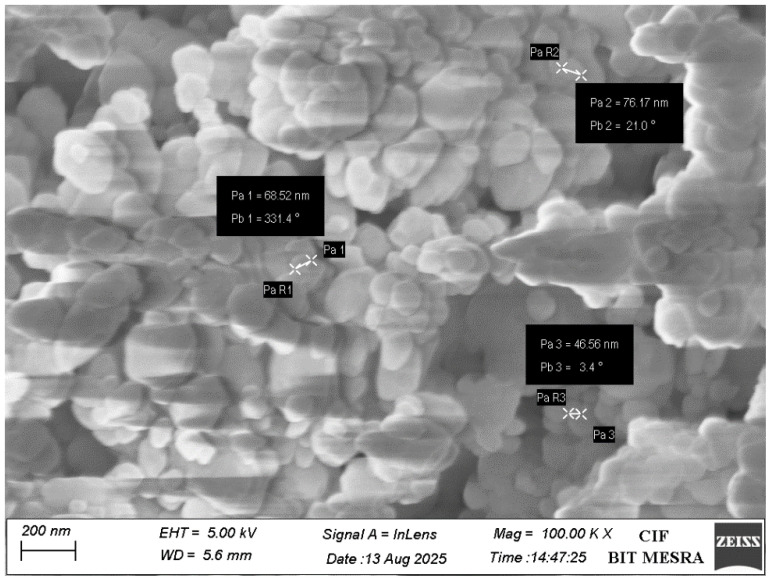
Diagrammatic representation of morphological analyses of CaO-NPs FESEM image, illustrating morphology with size. The scale bar and magnification of the FESEM image are 200 nm and 100,00×, respectively.

**Figure 4:**
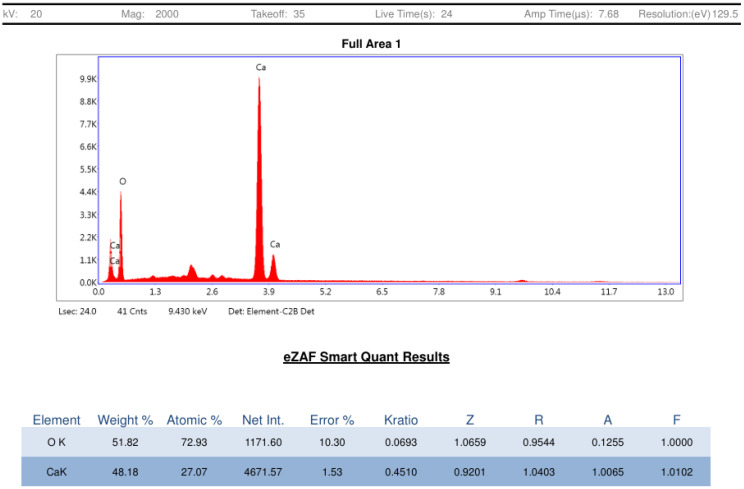
Graphical representation of elemental analysis of CaO-NP through EDS spectra showing weight percentage of calcium and oxygen present in the nanoparticles.

As illustrated in Figure 3, the morphological analysis was performed through FESEM. This analysis showed quasi-spherical particles with mean nanoparticle size 63.75 nm. Elemental analysis using energy-dispersive X-ray spectroscopy (EDX) confirmed the composition of CaO-NPs to be calcium (51.82%) and oxygen (48.18%), with no detectable impurities (Figure 4).

**Figure 5:**
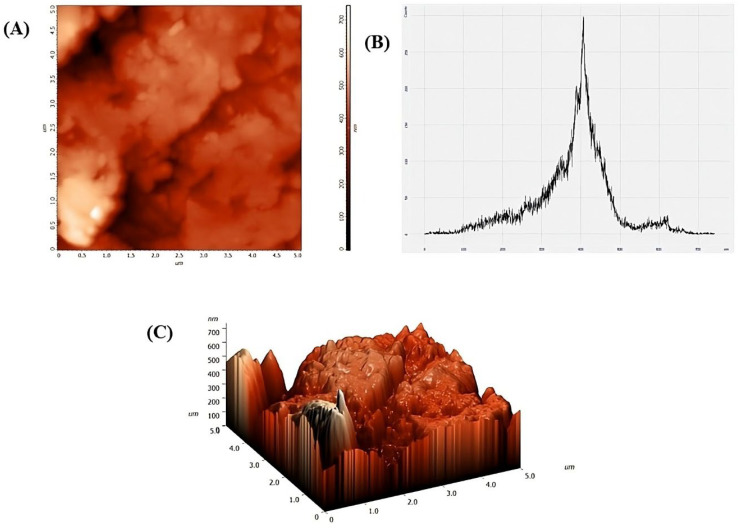
Visual representation of the surface properties of calcium oxide nanoparticles using Atomic Force Microscopy images: (**A**) 2D surface topography, (**B**) height profile, and (**C**) 3D surface topography.

Moreover, the AFM analysis provided detailed information about the surface morphology of the NPs as presented in Figure 5. The peak-to-peak value measured at 741.849 nm indicated the maximum height difference between the highest and lowest points on the NP surface. It provided a measure of the vertical roughness over the scanned area. Ten-point height value was recorded at 371.692 nm, which was an average of the five highest peaks and the five lowest valleys on the surface, giving a nuanced view of the roughness compared to a simple peak-to-peak measurement. The average roughness was 71.9299 nm. Root mean square (rms) roughness value was 99.1816 nm, which reflected the standard deviation of the surface height variations, indicating a relatively smooth surface with minor irregularities. A surface skewness value of −0.383964 indicated a slight predominance of valleys over peaks on the surface. A negative skewness suggested that the surface had more depressions than elevations.

### 3.2. Water Sample Physiochemical Characterization

Major water quality parameters were monitored throughout the experiment at the interval of 15 days (Table S1 in the Supplementary File). Over the course of 30 days, pH levels varied slightly between treatments (T1, T2, T3, PC, NC), with values ranging from 7.0 to 7.4, and continued to be stable within this range. Water temperatures were quite steady, ranging from 26.2 °C to 28.2 °C. Ammonia levels were found to be in the range of 0.13 to 0.26 mg/L. Nitrate levels were consistently low, ranging between 0.05 and 0.20 mg/L, with a slight increase by the 15th day. Dissolved oxygen exhibited values in between 6.1 and 6.7 mg/L and conductivity readings varied, commencing from 375 to 600 µS/cm and ranging from 417 to 522 µS/cm by the 30th day. Overall, all these recorded parameters denoted an ambient range suitable for *D. rerio* culture and experiments, indicating a stable environment.

### 3.3. Histological Analysis

**Figure 6:**
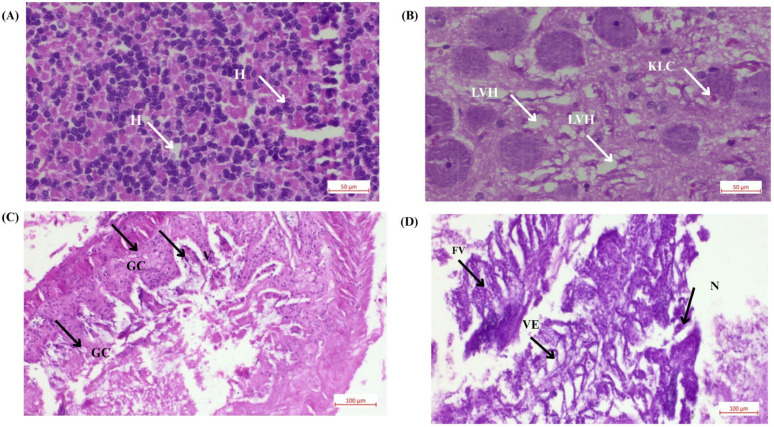
Histological analysis of *Danio rerio* tissues under the effects of CaO-NP-enriched diet: (**A**) Liver tissue from the negative control group showing normal anatomy. (**B**) Liver tissue from the T1 group (2.4 mg Cao-NPs/kg feed) showing pyknosis, degenerated hepatocytes, and numerous vacuoles, indicating severe damage and metabolic dysfunction (Note: H—hepatocyte, LVH—liver vacuolation hepatocyte, KLC—Kupffer-like cell; scale bar: 50 µm, magnification: ×400). (**C**) Intestinal tissue from the negative control group with normal villi and goblet cells. (**D**) Intestinal tissue from the T1 group (2.4 mg Cao-NPs/kg feed) showing vacuolization of enterocytes, hypertrophy of smooth muscles, pseudo crypts, and muscle layer degeneration, along with damaged goblet cells (Note: GC—goblet cell, FV—fused villi, VE—villar epithelium, N—necrosis, V—villi; scale bar: 100 µm, magnification: ×200).

The histological results revealed dose-dependent pathological changes across different tissue types. All the changes observed in the tissue sample after delivering the nanocalcium feed are presented in Figure 6.

Liver tissue analysis: The liver is a major organ for detoxification and metabolism in *D. rerio* [38]. The experimental groups (T1—2.4 mg CaO NPs/kg feed) displayed signs of pyknosis, indicated by dense and compact nuclei within the hepatocytes. This nuclear condensation is a hallmark of cell apoptosis and suggested significant cellular stress. The T1 group, in particular, showed a higher prevalence of degenerated hepatocytes and numerous vacuoles, which are indicative of cell death and impaired liver function.

Intestinal tissue analysis: The intestinal tissue analysis revealed substantial damage in the T1 group (2.4 mg CaO NPs/kg feed), characterized by multiple damaged goblet cells. These cells, which are responsible for mucus secretion and protecting the intestinal lining, showed signs of structural compromise. Vacuolization and branching within the lamina propria were observed, along with the fusion and degeneration of villi. These morphological changes significantly impaired nutrient absorption and disrupt the normal function of the intestinal barrier [39].

### 3.4. CaO-NP Accumulation Analysis in D. rerio Tissues Using ICP-OES

**Figure 7:**
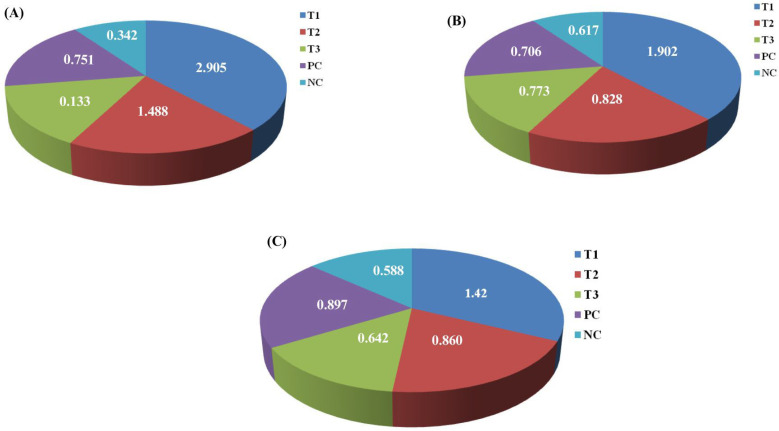
Pi-chart representation of calcium accumulation in various tissue samples across different experimental groups: (**A**) brain tissues; (**B**) intestinal tissues; (**C**) liver tissues. Highest concentration of calcium was observed in the T1 group tissue samples, indicating CaO-NP accumulation in all the organs. **Note:** All the different groups are represented using different colour codes; T1 is indicated by dark blue, T2 by red, T3 by green, the positive control by purple, and the negative control by cyan. NC = negative control; PC = positive control; T1 = Treatment 1 (2.4 mg CaO-NPs/kg feed); T2 = Treatment 2 (1.6 mg CaO-NPs/kg feed); T3 = Treatment 3 (0.8 mg CaO-NPs/kg feed).

The presence of calcium in the collected fish tissues was validated using the ICP-OES technique, as presented in Figure 7. The analysis showed notably higher calcium concentrations in the targeted organs associated with CaO-NP accumulation compared to the control groups. In the brain, calcium concentration was lowest in the negative control (0.342 mg/L), followed by the positive control (0.751 mg/L). Among the treatment groups, T1 had the highest calcium accumulation (2.905 mg/L), followed by T2 (1.488 mg/L) and T3 (0.133 mg/L). In the liver, calcium levels measured 0.588 mg/L and 0.897 mg/L in the negative and positive controls, respectively. Among the treatment groups, T1 showed the highest calcium concentration (1.42 mg/L), while T2 and T3 had lower values of 0.860 mg/L and 0.642 mg/L, respectively. In the intestine, calcium concentration was 0.617 mg/L in the negative control and increased to 0.706 mg/L in the positive control. Among the treatment groups, T1 exhibited the highest level (1.902 mg/L), followed by T2 (0.828 mg/L) and T3 (0.773 mg/L).

### 3.5. Feeding, Growth Performance, and Statistical Analysis in D. rerio

**Figure 8:**
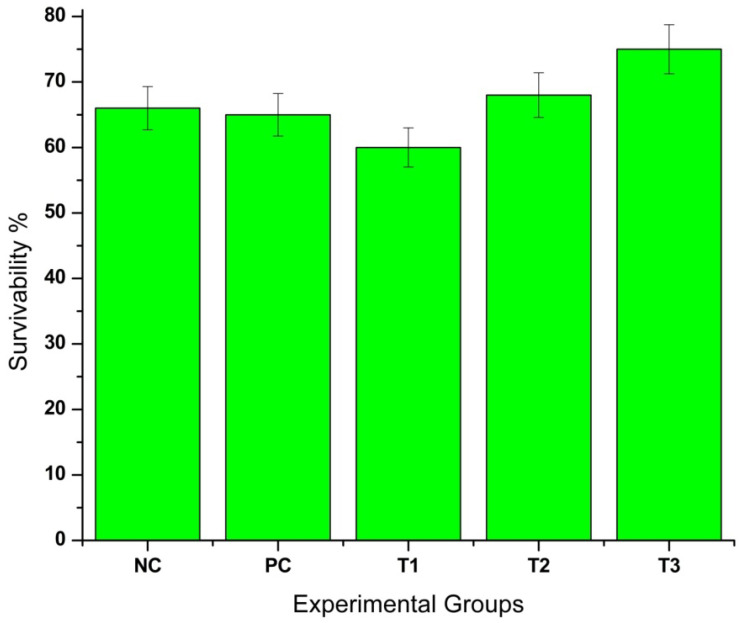
Graphical representation of survivability percentage of *Danio rerio* in all five experimental groups during the feeding period. Here, T3 (0.8 mg CaO-NPs/kg feed) and T1 (2.4 mg CaO-NPs/kg feed) groups showed highest and lowest survivability, respectively, signifying that 0.8mg/kg calcium oxide nanoparticle-infused fish diet provided more nourishment than the other experimental groups. Notes: NC—negative control; PC—positive control (2.4 mg); T1—2.4 mg CaO-NPs/kg feed; T2—1.6 mg CaO-NPs/kg feed; T3—0.8 mg CaO-NPs/kg feed.

As depicted by Figure 8, survivability percentage was one of the major parameters used to assess growth performance in *D. rerio*. It revealed distinct variations in survivability across different treatment groups. The T3 group (0.8 mg CaO-NPs/kg of feed) exhibited the most favourable outcomes, with the highest survivability rate (75%). In contrast, T1 (2.4 mg CaO-NPs/kg of feed) showed the lowest survivability (60%) and T2 demonstrated a moderate survivability rate (68%), while the positive control and negative control groups maintained relatively stable survival percentages of 65% and 66%, respectively.

**Figure 9:**
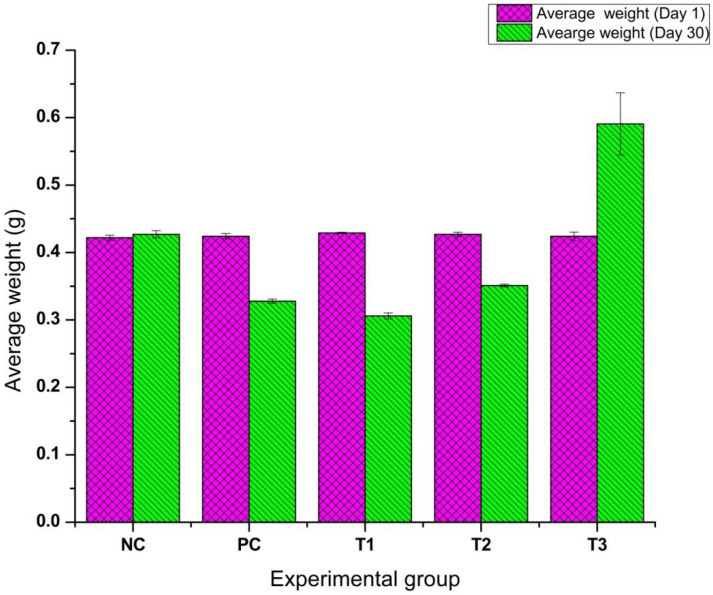
Graphical representation of *Danio rerio* average weight variation over 30-day time period across experimental feeding groups exposed to various concentrations of calcium oxide nanoparticles. Notes: NC—negative control; PC—positive control; T1—2.4 mg CaO-NPs/kg feed; T2—1.6 mg CaO-NPs/kg feed; T3—0.8 mg CaO-NPs/kg feed.

Similarly, Figure 9 shows the graphical representation of weight variation percentage in all the different experimental groups. It revealed distinct variations in weight change across different treatment groups. The T3 group (0.8 mg CaO-NPs/kg of feed) exhibited significant weight gain (+39.31%), indicating its effectiveness in promoting growth. In contrast, T1 (2.4 mg CaO-NPs/kg of feed) showed the most severe weight loss (−28.67%). Among the remaining groups, T2 demonstrated a weight reduction of −17.82%, while the positive control and negative control groups showed weight changes of −22.84% and +1.18%.

One-way ANOVA was conducted to analyze the effect of different treatments on the average weight of fish. The analysis revealed a statistically significant difference among the groups (F = 15.89, *p* < 0.0001), indicating that at least one treatment had a significant impact on fish weight. Post hoc Tukey’s test showed that the T3 group exhibited a significantly higher weight gain compared to all other groups (*p* < 0.05), suggesting its effectiveness in promoting growth. In contrast, T1 showed the lowest weight, indicating a toxic effect, while T2 also exhibited reduced weight but to a lesser extent than T1. No significant differences were observed between the negative control and positive control groups. These findings suggest that T3 is the most effective treatment for improving fish growth, while T1 and T2 negatively impact body weight.

### 3.6. Computational Analysis

#### 3.6.1. Selection of Metabolic Pathway

**Figure 10:**
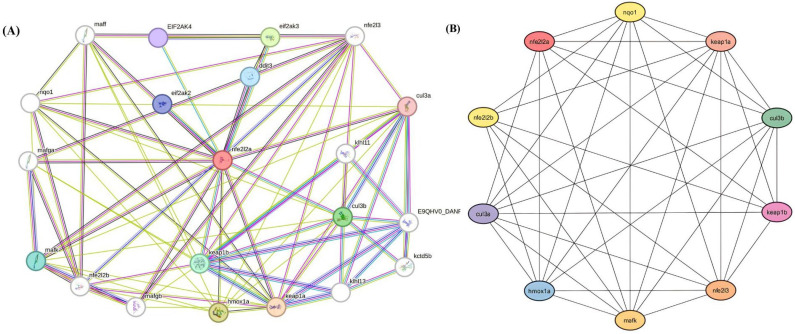
Pictorial representation of (**A**) complete protein–protein interaction and (**B**) major hub genes critically involved in NRF2 pathway for protein processing in endoplasmic reticulum in *D. rerio* using STRING database and Cytoscape.

The protein–protein interaction and hub gene identification was performed by Cytoscape (Version-3.10.3) and the STRING database (Version-12), as shown in Figure 10. These genes play a crucial role in regulating oxidative stress, detoxification, and cellular defence mechanisms. Among them, Keap1a exhibited the highest connectivity (degree = 9), highlighting its central role in NRF2 regulation. Keap1a is a key negative regulator of NRF2 and controls its stability through ubiquitination and degradation. Other highly connected genes, including Mafk, Cul3a, Cul3b, Nfe2l3, Hmox1a, and Nqo1 (degree = 8), were also identified as essential components of the NRF2 regulatory network, contributing to transcriptional activation, antioxidant defence, and cellular detoxification. Additionally, Nfe2l2a, Keap1b, and Nfe2l2b (degree = 6–7) demonstrated moderate connectivity, indicating their involvement in NRF2-mediated gene expression as secondary regulators. The interactions of these hub genes and their encoded proteins with CaO-NPs were analyzed to understand their influence in the NRF2 pathway. Understanding these interactions is crucial for evaluating the molecular response of CaO-NP-enriched feed in *D. rerio*, particularly in relation to growth, physiological adaptation, and oxidative stress regulation. Nrf2 is a key regulator of oxidative stress and apoptosis in *D. rerio*, interacting with proteins like Keap1a, Cul3a, Cul3b, nfe2I2b, and Nqo1 to maintain redox homeostasis. Through the Keap1-Nrf2 axis, it activates antioxidant genes during stress, mitigating ROS-induced damage and preventing apoptosis, as highlighted by STRING and Cytoscape network analyses.

#### 3.6.2. Three-Dimensional Protein Modelling and Verification

Protein modelling and the Ramachandran plot predict a protein’s 3D structure and assess its quality by analyzing dihedral angles, ensuring stability, and detecting structural errors. Organ-specific protein models were evaluated using DOPE scores and Ramachandran plots. For intestine glycogen, model 1 (DOPE score: −41623.52344) showed 86.3% favourable residues and 1.1% disallowed. For two brain proteins, dopamine and acetylcholinesterase, model 2 (−46333.44531) had 73.9% favourable and 3.2% disallowed residues and model 1 (−72319.20313) exhibited 89.7% favourable residues and only 0.2% disallowed, respectively. Liver catalase model 1 (−52376.73438) showed the highest quality with 91.6% favourable and 0.2% disallowed residues (Tables S3 and S4 in the Supplementary File). These results demonstrate the value of DOPE scores and Ramachandran plots in ensuring structural quality and functional reliability of protein models (Figure S2).

#### 3.6.3. Molecular Docking and Post-Docking Analysis of Proteins

Exposure to CaO-NPs induces excessive reactive oxygen species (ROS), overwhelming antioxidant defences and triggering apoptosis through ER stress and mitochondrial dysfunction [40]. Key pathways involve ATF4 and DDIT3 (CHOP) activation, while NRF2 suppression exacerbates oxidative damage. Persistent ROS disrupt the unfolded protein response (UPR) and proteasomal degradation, further driving cytotoxicity (Figure S1, Supplementary File).

**Figure 11:**
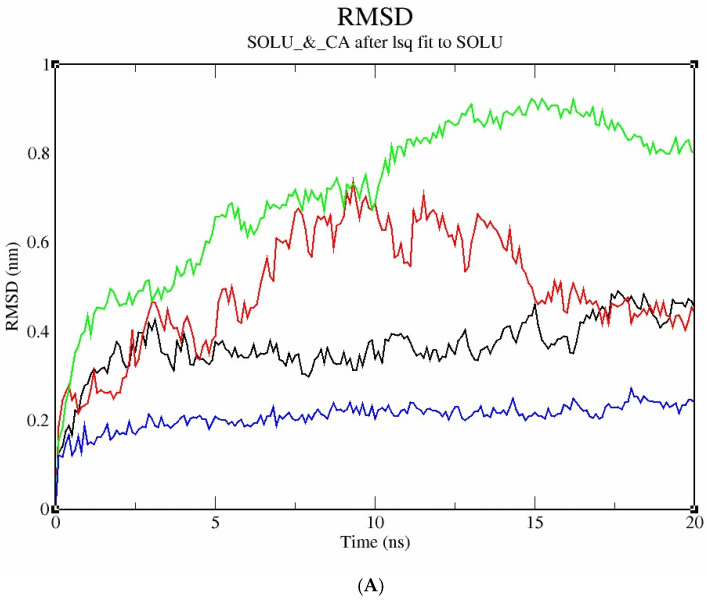

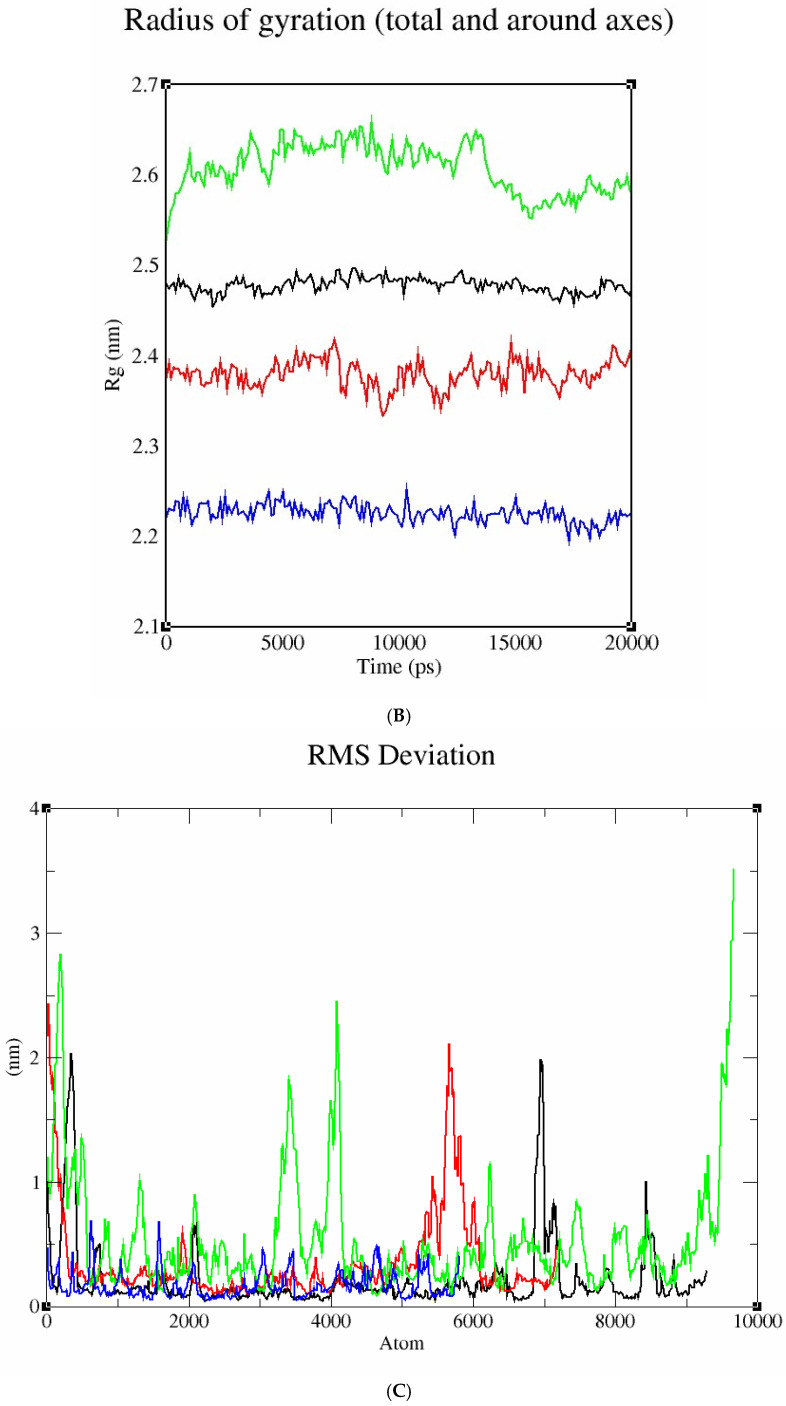
(**A**) Graphical representation of molecular dynamic study for modelled protein stability shown through RMSD profile of the modelled protein structures. The colour index used to represent the different protein structures is acetylcholinesterase, shown in black; catalase in red; dopamine in green; and glycogen in blue. (**B**) Graphical representation of molecular dynamic study for modelled protein stability showing radius of gyration of the modelled protein structures. The colour index used to represent the different protein structures is acetylcholinesterase in black, catalase in red, dopamine in green, and glycogen in blue. (**C**) Graphical representation of molecular dynamic study for modelled protein stability showing RMS deviation of the modelled protein structures. The colour index used to represent the different protein structures is acetylcholinesterase in black, catalase in red, dopamine in green, and glycogen in blue.

Figure 11A illustrates the molecular dynamic simulation analysis of four modelled protein structures, acetylcholinesterase (black), catalase (red), dopamine receptor (green), and glycogen phosphorylase (blue), to evaluate their structural stability and flexibility. The RMSD profile (Figure 10A) revealed that glycogen phosphorylase exhibited the lowest deviation (~0.2 nm), indicating high structural stability, while the dopamine receptor showed the highest RMSD (~0.8 nm), suggesting greater conformational flexibility.

The radius of gyration (Rg) plot (Figure 11B) further supported this, with glycogen phosphorylase displaying the most compact structure (~2.2 nm), and the dopamine receptor having the highest Rg (~2.6–2.7 nm), indicative of a more expanded conformation.

RMS fluctuation analysis (Figure 11C) showed minimal atomic deviations in glycogen phosphorylase, whereas the dopamine receptor exhibited pronounced fluctuations, especially in loop regions. Collectively, these results suggest that glycogen phosphorylase is the most stable and compact protein among the four, while the dopamine receptor is the most flexible, which may relate to its dynamic functional role. Glycogen, acetylcholinesterase, dopamine, and catalase showed strong binding, with structural stability confirmed via Root Mean Square Deviation (RMSD) and radius of gyration (Rg) analyses.

Molecular docking analysis revealed significant interactions between CaO-NPs and critical proteins essential for physiological functions in *D. rerio*. The binding interactions of various proteins with CaO-NPs were analyzed across several organs using Vina scores, cavity volume, no. of H-bonds, and contact residues as mentioned in Table 1. For Keap1a, the docking exhibited a Vina score of –3.5 with a cavity size of 1594 Å^3^, forming four hydrogen bonds. The interacting residues included GLY337, LEU338, GLY339, ALA340, ASN387, ARG388, VAL389, GLY390, VAL391, VAL401, GLY402, GLY403, ARG433, LEU434, GLY435, ALA436, GLY437, VAL438, GLY482, LEU483, GLY484, VAL495, SER528, ALA529, HIS530, GLY531, VAL532, SER575, GLY576, MET577, GLY578, and VAL579. The MafK protein displayed a Vina score of –2.4 with a cavity size of 439 Å^3^, establishing four hydrogen bonds with residues GLY21, GLU22, ASN23, ALA24, PRO25, ALA26, LEU27, GLN42, HIS43, LEU44, ARG45, GLY46, LEU47, THR48, GLU50, ASP51, VAL52, ARG54, LEU55, and ARG58. Similarly, Keap1b demonstrated a Vina score of –3.3 and a cavity size of 943 Å^3^ with four hydrogen bonds. The residues involved in interactions were GLY329, LEU330, ALA331, ALA332, CYS333, ILE381, GLY382, VAL383, GLY384, GLY427, VAL428, GLY429, VAL430, VAL440, GLY441, GLY442, ARG472, SER473, GLY474, ALA475, GLY476, SER520, ALA521, LEU522, GLY523, VAL524, SER567, GLY568, VAL569, GLY570, VAL571, and ALA572. Catalase protein exhibited the strongest binding with a Vina score of –3.7 and a cavity size of 1442 Å^3^, accompanied by four hydrogen bonds. The binding interface included residues ASN5, TYR17, LEU18, THR19, VAL20, LEU21, HIS46, PRO48, ASN60, ILE61, SER62, TYR63, PRO64, LYS65, GLN66, GLU67, LEU69, LEU70, GLU71, VAL72, LYS73, ASP110, ALA111, TRP112, SER113, SER146, PHE147, SER148, THR149, CYS150, ASP151, PRO152, HIS153, ASP154, GLY160, THR161, VAL162, PRO199, PRO201, THR224, THR225, TYR226, ARG325, VAL328, TYR330, ARG340, ASP341, SER342, SER343, and GLY344. Collectively, these results indicated stable ligand–protein complexes with multiple hydrogen bonds and favourable docking scores, suggesting that CaO-NP can effectively interact with antioxidant- and stress-related proteins, thereby influencing redox regulation pathways. It can potentially induce oxidative stress and apoptosis and inhibit the cellular cycle when higher concentrations of CaO-NPs accumulate in the respective organs (Figures S3 and S4, Supplementary File).

**Figure 12:**
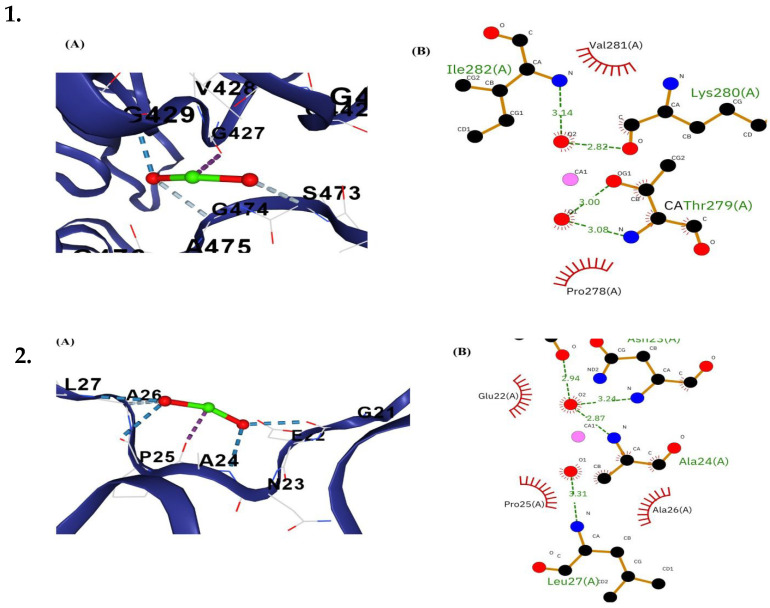
Diagrammatic representation of visualization of post-docking receptor–ligand complex of (**1**). mafk and (**2**). keap1b proteins of *Danio rerio.* (**A**) 3D representation of the calcium oxide nanoparticles binding within the peptide chain composed of different amino acid residues. Multiple hydrogen bonds are shown as blue dashed lines, while red dashed lines represent other polar or electrostatic interactions. (**B**) 2D interaction diagram showing the amino acid residues interacting with the ligand. Red arcs with spokes represent hydrophobic contacts, and green dashed lines denote hydrogen bonds along with their bond lengths (Å).

Figure 12 shows the molecular docking analysis of CaO-NPs and major metabolic proteins of *D. rerio*, including the post-docking interaction analysis of CaO-NPs with peptide chains of *D. rerio* mafk and keap1b proteins. Panel (A) displays the 3D visualization of the receptor–ligand complex, showing the CaO-NPs (green and red spheres) embedded within the peptide chain comprising residues Gly21 to Leu27. The blue dashed lines indicate multiple hydrogen bonds formed between the CaO-NPs and surrounding residues, while red dashed lines represent additional polar or electrostatic interactions stabilizing the complex. Panel (B) illustrates the 2D interaction map, identifying key residues involved in ligand binding. Hydrogen bonds are shown as green dashed lines with corresponding bond lengths (2.87–3.31 Å), and hydrophobic contacts are marked by red arcs with spokes. Notable interacting residues include Asn23, Ala24, and Leu27 forming hydrogen bonds, while Glu22, Pro25, and Ala26 contribute to hydrophobic interactions, suggesting a stable and specific binding interface between the nanoparticle and the protein active site.

**Figure 13:**
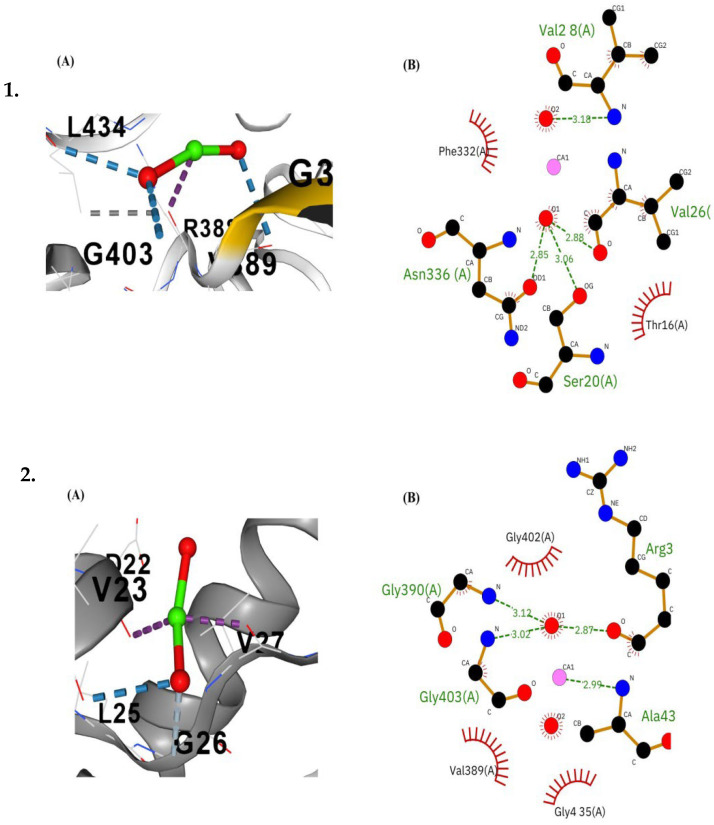
Diagrammatic representation of visualization of post-docking receptor–ligand complex in organ-specific proteins (**1**). Catalase and (**2**). Keap1a. (**A**) 3D representation of the calcium oxide nanoparticles binding within the peptide chain composed of different amino acid residues. Multiple hydrogen bonds are shown as blue dashed lines, while red dashed lines represent other polar or electrostatic interactions. (**B**) 2D interaction diagram showing the amino acid residues interacting with the ligand. Red arcs with spokes represent hydrophobic contacts, and green dashed lines denote hydrogen bonds along with their bond lengths (Å).

Figure 13 illustrates the post-docking interaction of CaO-NPs with catalase and keap1a proteins. Panel (A) of both complexes shows the 3D visualization of the CaO-NPs (green and red spheres) embedded within the peptide chains, interacting with nearby amino acid residues. Multiple hydrogen bonds (blue dashed lines) and polar or electrostatic interactions (red dashed lines) stabilize the binding environment. In the catalase complex (1A), key residues such as R38, G403, and L434 contribute to ligand stabilization. In complex 2A, residues like D22, V23, and G326 are involved in ligand binding. Panel (B) presents the 2D interaction diagrams, where green dashed lines indicated hydrogen bonds with distances ranging from 2.85 to 3.18 Å, and red arcs represent hydrophobic interactions. In complex 1B, residues such as Asn336, Ser20, and Val28 form strong hydrogen bonds, while Phe332 and Thr16 contribute hydrophobic interactions. In complex 2B, residues Gly390, Gly402, Gly403, and Ala43 form a network of hydrogen bonds, and hydrophobic contacts are seen with Val389 and Gly435. These interactions suggested a stable binding interface between the CaO-NPs and the target proteins.

## 4. Discussion

The current study investigated the dose-dependent analysis of CaO-NP-enriched fish feed for sustainable aquaculture, emphasizing their effects on *physiological growth and health status in D. rerio*. The CaO-NPs were chemically synthesized and characterized through multiple physicochemical techniques. DLS revealed a hydrodynamic diameter of 91.2 nm and a PDI of 0.215, indicating a narrow size distribution and good dispersibility in aqueous suspension. The hydrodynamic diameter measured by DLS conventionally exceeds core sizes observed under dry conditions, as it includes solvation layers and is influenced by larger aggregates. FESEM morphological analysis confirmed quasi-spherical nanoparticles with a mean core size of approximately 63.8 nm [41]. The moderate zeta potential of +12.0 ± 0.9 mV suggests electrostatic stabilization adequate to mitigate aggregation in suspension, which is essential for maintaining colloidal stability and consistent bioavailability. EDX analysis showed a composition of ~51.82% Ca and ~48.18% O, with no detectable impurities. XRD patterns displayed sharp peaks at 2θ ≈ 32°, 37°, 54°, and 67°, matching the cubic CaO phase (JCPDS No. 37-1497). Crystallite sizes estimated via the Scherrer equation ranged between ~6.6 and ~47.8 nm, with the most intense peak at ~32.23°, and absence of amorphous peaks verifying high crystallinity. FTIR analysis identified an O–H stretching band at ~3645 cm^−1^, a bending mode at 1631 cm^−1^, carbonate stretching at 1456 cm^−1^, a C–O stretching band at 1082 cm^−1^, and Ca–O vibrational modes at 872 and 618 cm^−1^, unequivocally confirming the presence of CaO and characteristic surface functional groups.

However, to understand the topographical insights of CaO-NPs, AFM analysis was performed. The physical basis behind this technique lies in its ability to probe surface interface behaviour, which plays a crucial role in nanoparticle–cell interactions and subsequent bioactivity. AFM operates by scanning a sharp nanoscale tip across the sample surface to detect interatomic forces, generating high-resolution topographical maps. The resulting height data reveals surface features such as roughness, particle distribution, and aggregation. These surface properties are essential for understanding CaO-NP stability, interaction potential, and biological compatibility. AFM results showed a peak-to-peak height of 741.849 nm and a ten-point height of 371.692 nm, suggesting moderate surface roughness. The average surface roughness was 71.93 nm, with an RMS roughness of 99.18 nm, supporting the notion of a relatively smooth surface conducive to biological interactions. Negative surface skewness (−0.384) indicated that the surface had more valleys than peaks, implying potential binding sites for cellular structures [42,43]. The moderate roughness and predominance of valleys on CaO-NP surfaces may provide binding niches that influence protein interaction and cellular uptake in *D. rerio*, potentially enhancing bioactivity while maintaining biocompatibility.

ICP-OES analysis demonstrated accumulation of calcium in *D. rerio* tissues upon exposure to CaO-NPs, with the brain exhibiting the highest accumulation, followed by intestine and liver. In the brain tissue, calcium concentration was lowest in the negative control (0.342 mg/L), followed by the positive control (0.751 mg/L). T1 showed the highest accumulation (2.905 mg/L), followed by T2 (1.488 mg/L) and T3 (0.133 mg/L). In the liver, calcium levels were 0.588 mg/L (negative control) and 0.897 mg/L (positive control); T1 recorded 1.42 mg/L, T2 0.860 mg/L, and T3 0.642 mg/L. In the intestine, calcium was 0.617 mg/L (negative control), 0.706 mg/L (positive control), with T1 showing 1.902 mg/L, T2 0.828 mg/L, and T3 0.773 mg/L. This organ-specific accumulation indicates dose-dependent nanoparticle bioavailability, potentially mediated by surface charge and nanoparticle size.

Histopathological analysis revealed tissue damage in the liver and intestine including hyperemia, hemorrhage, necrosis, villi degeneration, and cellular vacuolization. These findings indicated oxidative stress and disruption of cellular processes as mechanisms underlying high-dose toxicity. In the liver, hepatocytes in the T1 group (2.4 mg/kg CaO-NPs) exhibited pyknosis, characterized by nuclear condensation, which is a characteristic of apoptosis. In the intestine, goblet cell degeneration, vacuolization, and villi fusion in the T1 group indicated severe damage to the intestinal barrier. Goblet cells play a crucial role in mucus secretion, and their loss compromises intestinal protection. Structural disintegration of the villi reduces nutrient absorption, while increased permeability facilitates nanoparticle translocation into systemic circulation [44]. A lower dose (T3-0.8 mg CaO-NPs/kg feed) significantly enhanced *D. rerio* growth and body weight, showing CaO-NPs induced nutrient transport and metabolic efficiency in *D. rerio*. The T3 group demonstrated the highest survivability (75%) and weight gain (+39.31%), supporting its role in promoting growth. In contrast, T1 exhibited the lowest survivability (60%) and the most severe weight loss (−28.67%), suggesting toxicity at higher concentrations. T2 showed moderate survivability (68%) with weight reduction (−17.82%), while positive and negative control groups showed survival percentages of 65% and 66%, respectively, with weight changes of −22.84% and +1.18%. ANOVA results confirmed a statistically significant effect of treatments on weight gain (F = 15.89, *p* < 0.0001), with T3 showing the most beneficial outcomes. These results indicated that while lower doses of CaO-NPs enhance growth, higher concentrations negatively affect survivability and development, necessitating dose optimization for safe aquaculture applications.

These findings align with earlier research on other metal nanoparticles in aquaculture. For example, Zn-NP and Ag-NP incorporation in fish feed showed concentration-dependent effects on fish. In *Cyprinus carpio*, dietary supplementation with ZnSO_4_ and organic zinc at 40–60 mg Zn/kg feed improved growth, feed efficiency, and antioxidant enzyme activities, while 100 mg/kg showed no further benefits [45]. A separate 56-day study in common carp using ZnSO_4_ and ZnO-NPs at 0, 30, 100, and 500 mg/kg revealed that 500 mg/kg induced oxidative stress, reduced condition factor, and caused zinc accumulation in liver and kidney [46]. In grass carp (*Ctenopharyngodon idella)*, 30 mg Zn/kg feed provided through ZnSO_4_, ZnO-NPs, zinc lactate, or zinc glycine improved growth, but organic forms (Zn-Gly, Zn-Lac) significantly enhanced intestinal villus height and tight junction integrity; ZnO-NPs caused mild intestinal inflammation [47]. Additionally, a 60-day trial in rainbow trout (*Oncorhynchus mykiss*) with Ag-NPs at 0.5–4.0 mg/kg feed indicated that ≥2.0 mg/kg reduced growth, impaired liver and gill structure, and induced oxidative stress, while ≤1.0 mg/kg was relatively safe [48].

To support these observations, an in silico study was conducted to explore protein–nanoparticle interactions and their molecular mechanisms. The NRF2 signalling pathway plays a pivotal role in regulating oxidative stress, cellular detoxification, and defence mechanisms [49]. In this study, hub gene analysis revealed that Keap1a had the highest degree of connectivity (degree = 9), indicating its central function as a key negative regulator of NRF2. Other hub genes such as Mafk, Cul3a, Cul3b, Nfe2l3, Hmox1a, and Nqo1, each with a connectivity degree of 8, were also identified as critical contributors to the NRF2 pathway. These genes are involved in transcriptional activation, antioxidant defence, and the cellular detoxification process. Additionally, genes like Nfe2l2a, Keap1b, and Nfe2l2b showed moderate degrees of connectivity (6–7), suggesting their role as secondary regulators in NRF2-mediated responses. These interactions suggest that CaO-NPs modulate NRF2-mediated cellular responses, either promoting redox balance at lower doses or triggering oxidative damage at higher concentrations [50]. To assess the structural quality of key proteins, 3D protein modelling was performed and validated using DOPE scores and Ramachandran plots. These results confirmed the structural reliability of the models and support their use in docking analysis. Additionally, the docking results provided molecular-level evidence for nanoparticle-driven physiological responses, correlating with the observed histopathological and growth outcomes. Post-docking visualization confirmed that proteins such as catalase, keap1b, keap1a, and mafk exhibited the strongest binding affinities with CaO-NPs through multiple hydrogen bonds. These findings signified that CaO-NPs interact variably with proteins across different organs, suggesting their potential involvement in nanoparticle-induced physiological or stress-related responses [51,52,53].

Collectively, the present study highlights that CaO-NPs have a strong affinity for neural tissues, potentially due to their ability to cross biological barriers such as the blood–brain barrier [54]. CaO-NPs enter the bloodstream through the digestive tract after being ingested in feed. Due to their nanoscale size and surface charge, they can circulate freely and interact with endothelial cell lining [55]. Once inside, CaO-NPs may bind to calcium transporters, disrupting normal calcium homeostasis. The excess calcium can stimulate neurotransmitter release and interfere with synaptic signalling. At low concentrations, CaO-NPs may enhance neural function by supporting synaptic activity. At high doses, excessive accumulation can cause oxidative stress, neuro-inflammation, and neuronal apoptosis [56]. The effects of CaO-NPs vary with dosage as lower doses may increase physiological activity, while higher concentrations may result in oxidative stress, apoptosis, and toxicity in critical organs because of the production of ROS. Nano-calcium holds significant promise for enhancing growth, nutrient absorption, and fish health at appropriate doses. Calcium’s critical role in muscle contraction, nerve function, and bone health makes its nanoscale delivery beneficial. Thus, while low-dose CaO-NPs may offer physiological benefits, higher concentration causes neurotoxic risks, emphasizing the need for optimized dosing in aquaculture. Collectively, these findings underscore the potential of CaO-NPs to significantly modulate molecular and physiological responses in *D. rerio* through direct interaction with key regulatory proteins. Such effects, particularly those linked to oxidative stress and apoptosis, emphasize the importance of controlled nanoparticle dosing in aquaculture settings to ensure safety and sustainable growth outcomes [57,58,59,60].

Future research should focus on determining safe dosage ranges, assessing long-term effects on fish health, and evaluating ecological impacts to ensure the sustainable application of nanotechnology in aquaculture. By understanding nanoparticle chemistry, their systemic effects on cellular components on aquaculture animals and their effective use can enhance aquaculture productivity while minimizing risks.

## 5. Conclusions

In the present study, CaO-NPs were successfully synthesized using the co-precipitation method, exhibiting a spherical morphology with a crystallite size of 47.8 nm and moderate stability (+12 mV), making them suitable for cellular interactions. Upon dietary administration in *D. rerio*, CaO-NPs exhibited dose-dependent bioaccumulation, with the intestine, liver, and brain showing the highest levels. ICP-OES analysis revealed the accumulation of CaO-NPs in critical organs, correlating with dose-dependent toxicity. Growth performance was significantly improved at 0.8 mg/Kg dose, while histological alterations were evident at higher doses. Computational analysis revealed the mechanistic insights behind CaO-NPs modulated antioxidant and detoxification pathways by interacting with organ-specific and NRF pathway proteins. It revealed that CaO-NPs modulate redox homeostasis primarily through activation of the NRF2-mediated antioxidant pathway, along with altered expression of stress-related genes. These findings suggested that CaO-NPs can promote beneficial growth responses but excessive accumulation may lead to toxicity and organ damage. Based on the findings, a dose of 0.8 mg CaO-NPs/kg feed is recommended for promoting growth, nutrient absorption, and physiological health in *D. rerio*, while avoiding adverse histopathological effects and mortality. These findings would contribute to a better understanding of the environmental safety and sustainable application of nanocalcium for fish health. Further research is needed to explore assess chronic exposure impacts and potential applications of CaO-NPs in aquaculture to ensure their safe and sustainable use.

## Figures and Tables

**Table 1:** Tabular representation of molecular docking energies of CaO-NPs with selected proteins in the form of Vina score, cavity volume, contact residues, and no. of H-bonds of each protein of *Danio rerio*.

Organs	Proteins	Cur Pocket ID	Vina Score	Cavity Volume (Å^3^)	Contact Residues	No. of H-Bonds
	Polo like kinase 1	C1	−3.3	1173	ARG43 LEU45 CYS53 ALA66 LYS68 VAL70 MET84 GLU87 ILE88 HIS91 VAL114 LEU116 GLU117 ILE118 CYS119 ARG120 ARG121 ARG122 SER123 ILE159 HIS160 ARG161 ASP162 LYS164 ASN167 PHE169 LEU170 GLY179 ASP180 PHE181 GLY182 LEU183 ALA184 THR200 PRO201 ASN202	-
Gills	TRPV6	C1	−3.2	3387	GLY194 VAL233 PRO234 ASN235 TYR236 GLY238 ARG247 SER283 ARG284 ASP286 GLU287 HIS288 SER289 GLU292 ILE293 ILE294 THR296 SER297 HIS298 VAL409 ARG413 PHE414 PHE415 GLN417 ALA419 LEU420 GLY421 GLY422 HIS425 TYR466 ARG469 GLN595 VAL596 ALA598 THR599 LEU601 MET602 ARG605	-
	N-K ATPase alpha−2	C1	−3.2	4499	LYS89 GLN90 LEU91 PHE92 GLY93 GLY94 PHE95 SER96 GLY139 CYS140 GLN145 LYS148 SER149 SER150 ILE152 GLU273 VAL274 GLY275 THR277 LEU332 ALA343 LEU352 VAL353 LYS354 ASN355 LEU356 GLN700 ALA718 LEU719 LYS720 LYS721 ALA722 ASP723 ILE724 GLY725 GLN738 ALA739 ALA740 ASP741	-
Intestine	Glutaminase A	C1	−3.1	1811	SER252 LYS255 GLU278 PRO279 SER280 GLY281 LEU282 ARG283 PHE284 ASN285 LYS286 LEU287 PHE288 LEU289 HIS296 ASN297 MET299 VAL300 ASN301 ALA302 ARG353 ASN354 ILE357 TYR380 CYS384 SER428 CYS429 GLY430 MET431 TYR432 ASP433 SER435 LYS447 VAL450	-
	Glycogen	C1	−3.1	2601	GLY65 PHE67 LYS85 ARG96 GLU97 ILE100 HIS179 ARG180 ASP181 LYS183 ASN186 ASP200 PHE201 GLY202 SER203 ALA204 LYS205 ILE217	-
	Isotocin	C1	−2.6	581	ARG40 GLN41 CYS42 ARG50 GLY51 ARG52 CYS53 PHE54 CYS60 GLY61 GLU62 GLY63 ILE64 GLU87 MET88 SER89 GLY90 ARG98 CYS99 ALA100 ALA101 PRO102 GLY103 VAL104 CYS117 VAL118 ASP119 GLY120 ASP121 ALA122 ALA124 ALA125 ALA126	-
	Dopamine	C1	−3.1	2229	LE152 PHE153 PHE154 ILE155 ARG156 PRO190 GLU191 SER192 LEU193 HIS194 GLN195 VAL196 PHE200 SER201 ASP202 ARG203 MET284 TRP292TRP294 ASP298 THR300 LYS301 VAL302 TRP303 SER304 GLU307 PHE308 ASP437 ASP438 ASN439 VAL440 THR441 GLN442 VAL443 PHE446 LEU451 ALA454 GLU455 ARG456 ARG458 LEU459	-
Brain	Acetylcholineeasterase	C1	−3.1	1282	ALA437 GLN438 THR453 GLY455 TRP456 GLY457 ASN458 SER459 TYR464 ASN465 SER466 GLY467 ASN468 SER469 HIS470 GLY471 ALA472 VAL473 TYR474 GLY535 ASN536 PRO537 GLN553 PHE554 SER555 ALA556 ASN557	-
	GST	C1	−3.2	633	TYR8 VAL11 LYS12 GLY13 ARG14 CYS15 GLY16 ALA17 CYS48 VAL49 PHE50 GLY51 GLN52 LEU53 PRO54 LYS55 PHE64 GLN65 SER66 ASN67 ALA68 LEU70 ASN94 ASP95 VAL97 GLU98 ASP99 ARG101 TYR104 ILE105 TYR109 ASN153 ASP156 LEU157 ASN160 ILE202 ASN203 GLY204	2
Liver	Glutathione synthetase	C1	−3.3	2304	ARG125 ASP127 GLU144 ILE145 ASN146 THR147 ILE148 ALA149 ALA150 SER151 LEU209 VAL210 GLU211 GLN214 ASN216 HIS220 ARG267 ASN268 TYR270 LYS306 LYS365 PRO366 GLN367 ARG368 GLU369 GLY370 GLY371 GLY372 ASN373 ASN374 TYR376 LEU411 LEU412 ARG413 PRO414 SER421 ARG451 ASP459	2
	IGF-1	C1	−2.7	770	ARG65 TYR68 PHE69 SER70 LYS71 PRO72 THR73 SER82 HIS83 ASN84 ARG85 GLY86 ILE87 VAL88 ASP89 GLU90 ARG100 GLU102 MET103 TYR104 CYS105 ALA106 PRO107	-
	Catalase	C1	−3.7	1442	ASN5 TYR17 LEU18 THR19 VAL20 LEU21 HIS46 PRO48 ASN60 ILE61 SER62 TYR63 PRO64 LYS65 GLN66 GLU67 LEU69 LEU70 GLU71 VAL72 LYS73 ASP110 ALA111 TRP112 SER113 SER146 PHE147 SER148 THR149 CYS150 ASP151 PRO152 HIS153 ASP154 GLY160 THR161 VAL162 PRO199 PRO201 THR224 THR225 TYR226 ARG325 VAL328 TYR330 ARG340 ASP341 SER342 SER343 GLY344	4
	Keap 1a	C1	−3.5	1594	GLY337 LEU338 GLY339 ALA340 ASN387 ARG388 VAL389 GLY390 VAL391 VAL401 GLY402 GLY403 ARG433 LEU434 GLY435 ALA436 GLY437 VAL438 GLY482 LEU483 GLY484 VAL495 SER528 ALA529 HIS530 GLY531 VAL532 SER575 GLY576 MET577 GLY578 VAL579	4
	Mafk	C1	−2.4	439	GLY21 GLU22 ASN23 ALA24 PRO25 ALA26 LEU27 GLN42 HIS43 LEU44 ARG45 GLY46 LEU47 THR48 GLU50 ASP51 VAL52 ARG54 LEU55 ARG58	4
	Cul3a	C2	−3.1	1386	GLY571 ILE573 ALA586 LEU587 LEU588 THR589 GLY590 SER591 ASN592 THR593 ARG594 LYS595 HIS596 PHE671 THR672 SER673 LYS674 HIS676	2
	Cul3b	C2	−3.0	5731	GLU428 ARG429 LYS432 GLN433 CYS463 GLN464 PHE465 THR466 SER467 LYS468 LEU469 ASP475 ILE478 VAL506 LEU507 THR508 THR509 GLY510 TYR511 TRP512 PRO513 THR514 GLN515 SER516 GLN546 GLU681 PRO684 GLU685 ARG686 GLU688 THR689 LYS692 GLU700 ARG731	2
	Nfe2I3	C2	−3.1	1225	ILE17 LEU19 SER20 LEU21 ILE22 GLY23 VAL24 ARG25 HIS334 ILE335 ASN336 MET337 LEU338 ASP339 LEU340 PHE570 SER574 PHE578 PRO591 GLU592 TYR594 THR595 LEU596 HIS597 CYS598 GLN607 ARG609	-
	Keap1b	C2	−3.3	943	GLY329 LEU330 ALA331 ALA332 CYS333 ILE381 GLY382 VAL383 GLY384 GLY427 VAL428 GLY429 VAL430 VAL440 GLY441 GLY442 ARG472 SER473 GLY474 ALA475 GLY476 SER520 ALA521 LEU522 GLY523 VAL524 SER567 GLY568 VAL569 GLY570 VAL571 ALA572	4
	Nfe2I2b	C2	−2.5	1003	LEU143 ASP144 VAL145 LEU146 GLU147 SER148 GLU149 SER150 SER151 SER152 LEU153 GLU156 LYS448 ARG452 TYR455 PRO472 ARG490	1
	Nfe2I2a	C1	−2.3	889	ASN272 PHE275 TYR276 PRO277 GLU278 MET279 MET534 LYS535 GLN536 LEU538 SER539 THR540 TYR542 GLN543 PRO559 ASN560 GLU561 SER563 LEU564 GLN565 HIS566 ARG577	2
	NAD(P)H Quinone dehydrogenase 1	C5	−3.2	219	MET143 GLN144 ASN145 MET146 TYR147 ASP148 ASN149 TRP184 ASN188 ARG192 PHE237 ALA238 PRO239 SER240 PHE243 LEU245 THR269 HIS272	3

## Data Availability

Data can be provided on basis of request.

## Supplementary Materials

The following supporting information can be downloaded at: https://www.mdpi.com/article/10.3390/bioengineering12101016/s1. The supplementary file contains KEGG pathway maps (NRF2 signaling, protein processing, apoptosis), protein modeling and docking results (Ramachandran plots, 3D structures, 2D/3D docking interactions), ANOVA-based zebrafish weight variation, water quality parameters, diet formulation details, and organ-specific protein datasets related to calcium metabolism and NRF2 function. Overall, the file provides the computational models, docking insights, physiological data, and experimental conditions that substantiate the main manuscript findings.
