# Supplementary material for: Evaluating the Molecular Basis of Nanocalcium-Induced Health Regulation in Zebra Fish (Danio rerio)
Source: Bioengineering (Basel). 2025 Sep 24;12(10):1016. doi: 10.3390/bioengineering12101016 (PMC12561382; doi:10.3390/bioengineering12101016)
Supplement: Supplementary file 1 [file bioengineering-12-01016-s001.zip › bioengineering-3778473-supplementary.pdf]

Supplementary file for:

Evaluating the Molecular Basis of Nanocalcium-Induced Health Regulation in Zebra Fish (*Danio rerio*)

Madhubala Kumari[a], Aastha Tiwary[a], Rishav Sheel[a,b], Arnab Roy Choudhury[c], Dipak Maity[d,e*] , Biplab Sarkar[b*], Koel Mukherjee[a*]

[a] Department of Bioengineering and Biotechnology, Birla Institute of Technology, Mesra, Ranchi-835215, Jharkhand, India.

[b]School of Molecular Diagnostics, Prophylactics and Nanobiotechnology, ICAR-Indian Institute of Agricultural Biotechnology (IIAB), Garhkhatanga, Ranchi, Jharkhand, 834003, India.

[c]Downstream Agro-Processing Division, ICAR- National Institute of Secondary Agriculture (NISA), Namkum, Ranchi, Jharkhand, 834010, India.

[d]Integrated Nanosystem Development Institute, Indiana University Indianapolis, IN46202, USA

[e]Department of Chemistry and Chemical Biology, Indiana University Indianapolis, Indianapolis, IN46202, USA

Corresponding authors:

Dr. Biplab Sarkar (biplab_puru@yahoo.co.in)

Dr. Koel Mukherjee (koelmukherjee@bitmesra.ac.in)

Dr. Dipak Maity (dipakmaity@gmail.com)

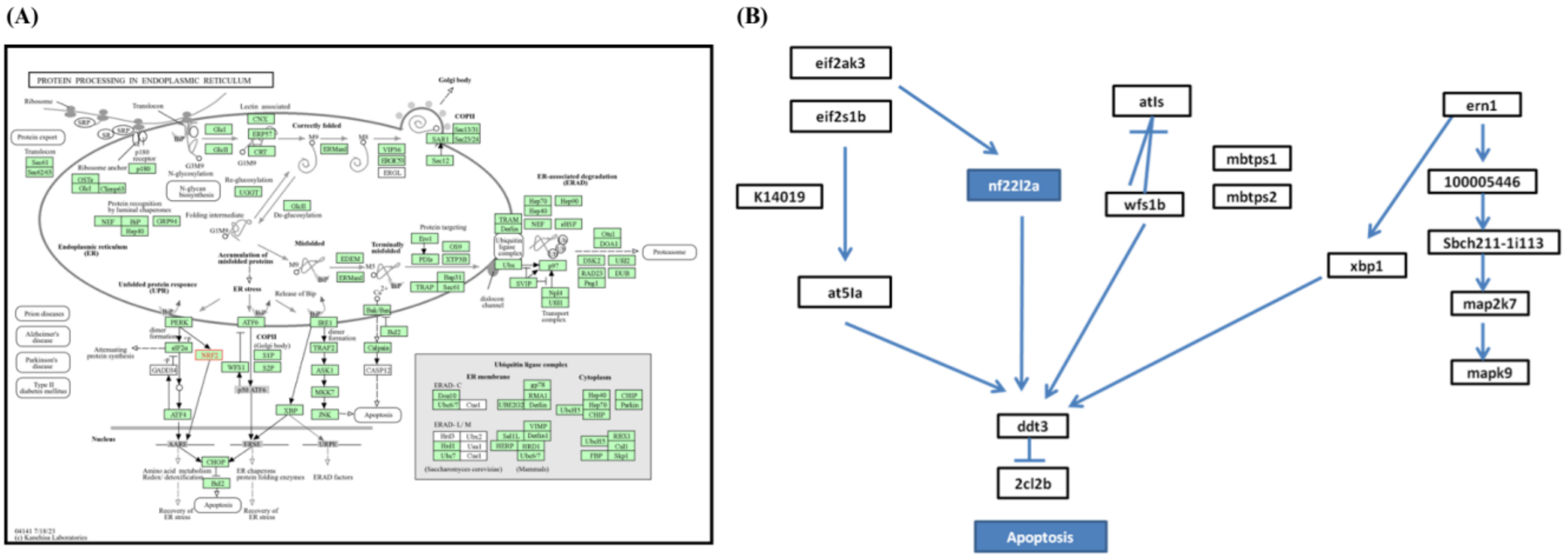

Figure S1. Pictorial representation of (A) complete NRF2 pathway for protein processing in endoplasmic reticulum in *Danio rerio* using KEGG pathway and (B) selected part of NRF2 pathway involved in apoptosis.

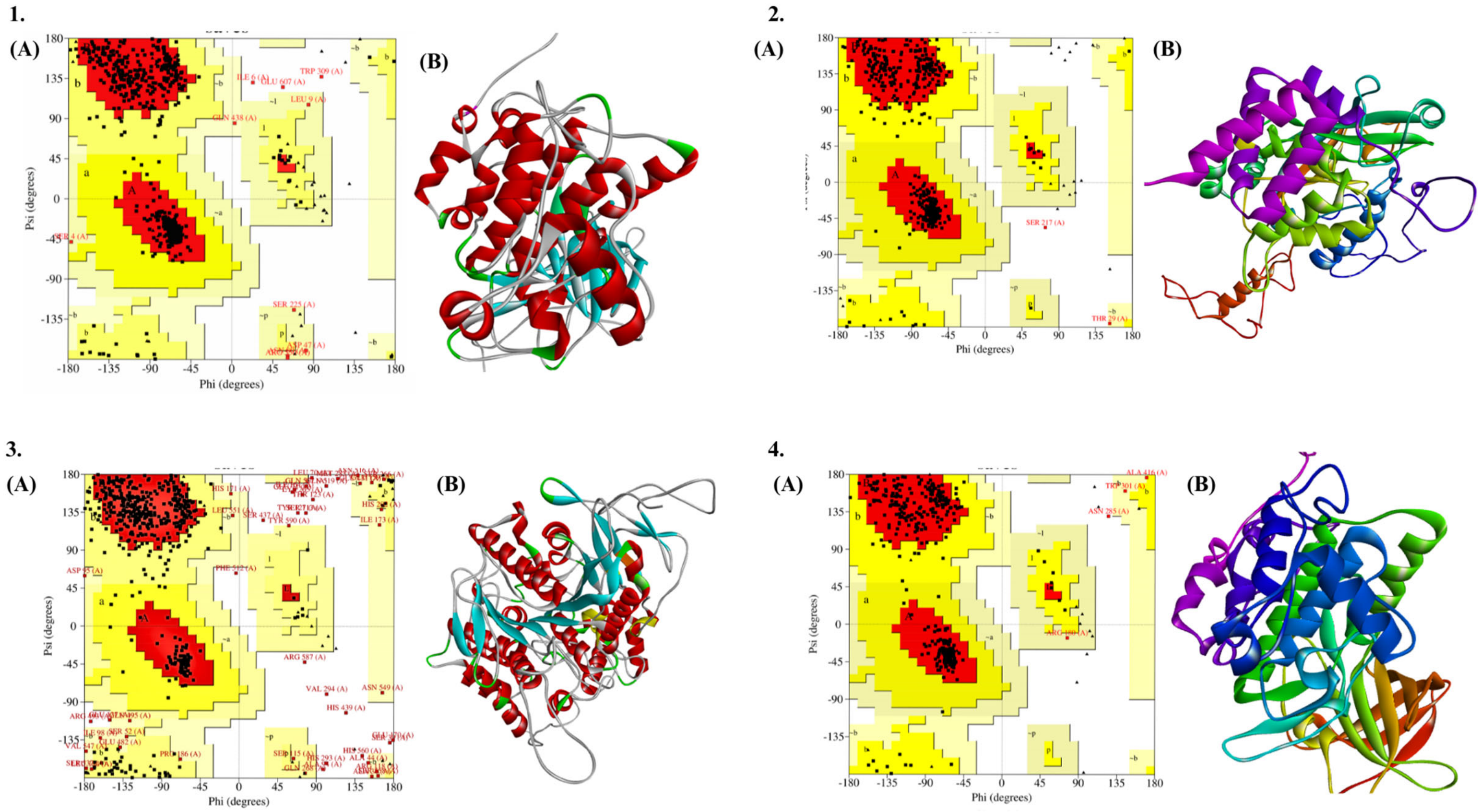

Figure S2. Pictorial representation of various modelled proteins, showing (A) Ramachandran plot of the modelled structure and (B) 3D protein structure models of (1.) Acetylcholineeasterase; (2.) Dopamine; (3.) Glycogen; and (4.) Catalase of *Danio rerio.*

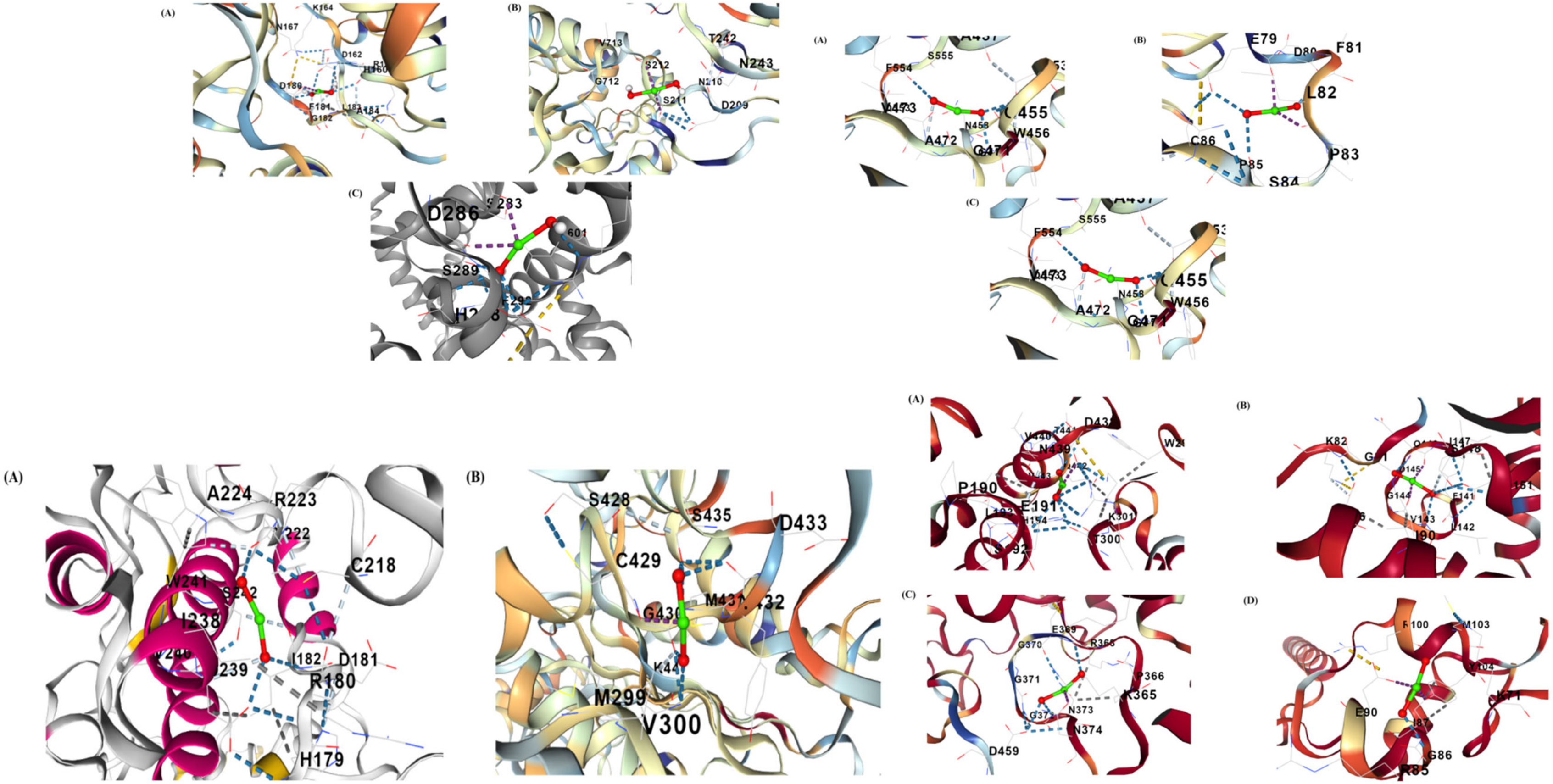

Figure S3. Diagrammatic representation of three-dimensional (3D) post-docking visualization of proteins with CaO-NPs showing multiple H-bonds in the form of dotted green lines. (1). (A) Isotocin (B). N-KATPase. (C). GST. (2). (A) Glutathionesynthetase. (B) IGF. (C) Glutaminase A. (3). (A) Heme Oxygenase 1a (hmox1a). (B) Small Maf Protein K (mafK). (C) Cullin 3b (cul3b). (D) NAD(P)H Quinone Dehydrogenase 1 (nqo1), showing strong interactions with the CaO-NPs.

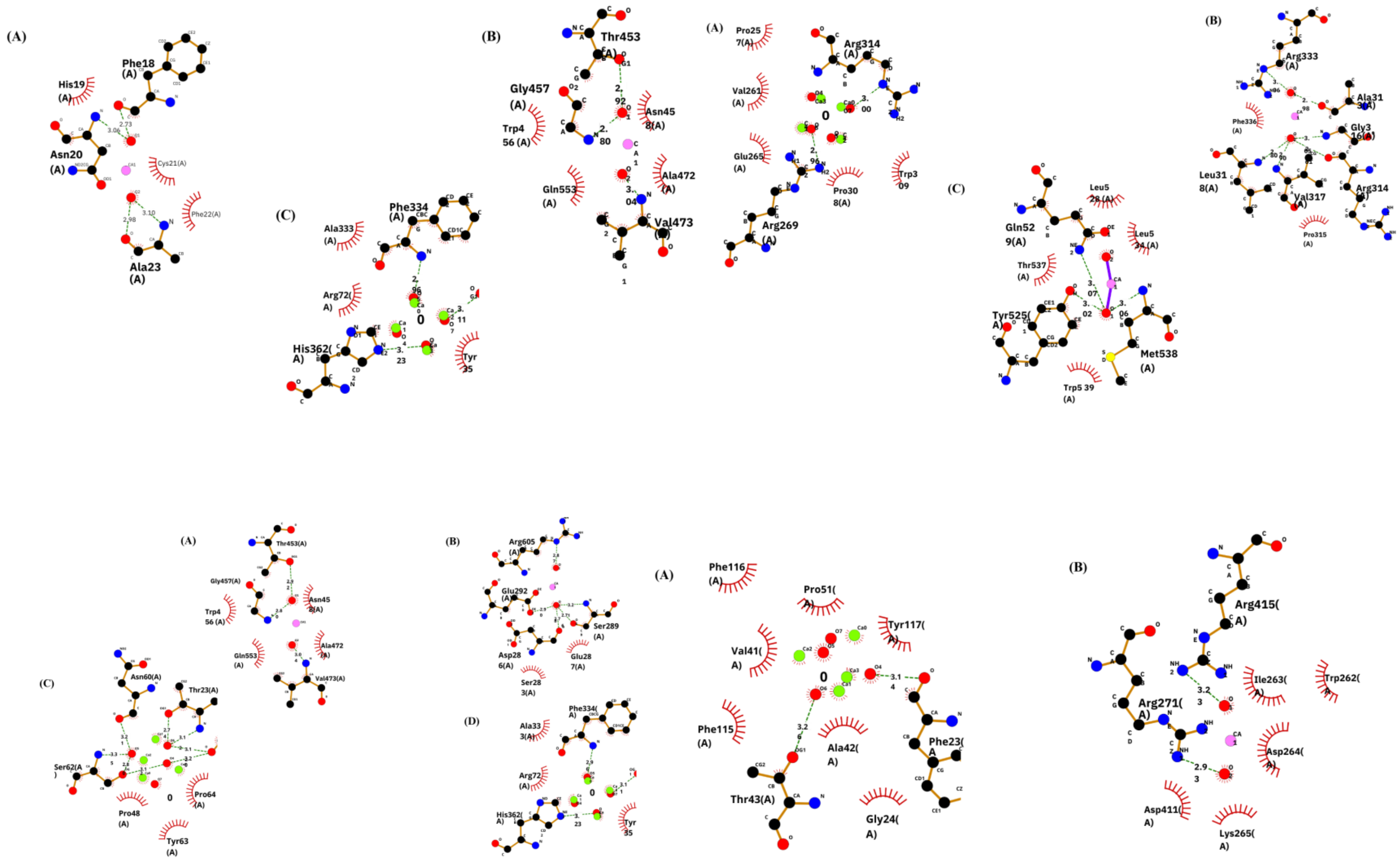

Figure S14. Diagrammatic representation of two-dimensional (2D) post-docking visualization of proteins with CaO-NPs showing multiple H-bonds in the form of dotted green lines. (1). (A) Isotocin. (B). N-KATPase. (C). GST. (2). (A) Glutathionesynthetase. (B) IGF. (C) Glutaminase A. (3). (A) Heme Oxygenase 1a (hmox1a). (B) Small Maf Protein K (mafK). (C) Cullin 3b (cul3b). (D) NAD(P)H Quinone Dehydrogenase 1 (nqo1)(4). (A), (B) Diagram showing maximum H-bonds, i.e., eight, five, six, and four in the cases of catalase and glutathione synthetase (liver); dopamine (brain); and PLK-1(gills), respectively, showing strong interactions with the CaO-NPs.

One Way ANOVA

Overall ANOVA

	DF	Sum of Squares	Mean Square	F Value	Prob>F
Model	3	0.82179	0.27393	79.63704	7.81499E-10
Error	16	0.05504	0.00344		
Total	19	0.87682			

Null Hypothesis: The means of all levels are equal.
Alternative Hypothesis: The means of one or more levels are different.
At the 0.05 level, the population means are significantly different.

Fit Statistics

	R-Square	Coeff Var	Root MSE	Data Mean
	0.93723	0.27883	0.05865	0.21034

Figure S5. Pictorial representation of spread of ANOVA used in the calculation of *Danio rerio* average weight variation over 30-day time period across experimental feeding groups exposed to various concentrations of calcium oxide nanoparticles.

Table S1. Tabular representation of water quality parameters obtained during experimental period starting from 0th day to 30th day.

0th day		15th Day					30th Day				
		T1	T2	T3	PC	NC	T1	T2	T3	PC	NC
**pH**	7.0±0.3	7±0.14	7.1±0.2	7.4±0.01	7.1±0.2	7.3±0.02	7.4±0.01	7.2±0.02	7.1±0.2	7.2±0.4	7.2±0.3
**Temperature (°C)**	26.2±2.3	27.21±2.1	26.6±2.2	26.2±2.4	27.3±2.3	27.4±2.1	26.3±2.2	27.3±2.4	28.2±2.4	27.3±2.1	26.4±2.3
**Ammonia (mg/L)**	0.19±0.04	0.13±0.2	0.23±0.3	0.26±0.2	0.13±0.4	0.23±0.2	0.25±0.13	0.23±0.2	0.26±0.2	0.23±0.6	0.42±0.2
**Nitrate (mg/L)**	0.06±0.03	0.16±0.3	0.20±0.2	0.06±0.03	0.13±0.01	0.11±0.04	0.16±0.1	0.20±0.3	0.18±0.03	0.19±0.04	0.05±0.01
**Dissolved Oxygen (mg/L)**	6.5±0.03	6.4±0.03	5.6±0.48	5.8±0.3	6.7±0.03	6.1±0.03	6.4±0.03	6.6±0.03	5.8±0.03	6.6±0.03	6.8±0.03
**Conductivity (µS/cm)**	425±35.5	500±23.3	375±35.35	500±23.4	600±45.3	400±20.34	435±49.49	417±24.7	426±12.7	421±5.61	522±137.8

Note: Each value is the mean±SD of the duplicates of each individual replica.

Table S2. Tabular representation of ingredient list for nutrient composition of the fish experimental diet.

**Ingredients**	**Treatment 1**	**Treatment 2**	**Treatment 3**	**Positive Control**	**Negative Control**
Wheat flour (g)	30	30	30	30	30
Ground nut cake (g)	15	15	15	15	15
Corn flour (g)	15	15	15	15	15
Fish meal (g)	25	25	25	25	25
Sunflower oil (mL)	10	10	10	10	10
Cod liver oil (mL)	2	2	2	2	2
Carboxymethyl cellulose (g)	3	3	3	3	3
Nano calcium oxide (mg/kg dry diet)	2.4	1.6	0.8	2.4	-

Note: Calcium nitrate was used as calcium source in positive control group.

Table S3. Tabular representation of details of organ-specific major proteins involved in calcium metabolism and their different templates and similarity percentages used for the protein modelling of query proteins of *D. rerio*.

Organ	Protein	Seq. length	NCBI Accession No.	Active site	Query PDB ID	Template	Percentage Identity (%)
**Gills**	Polo-like kinase 1	595	ADK12654	45- 68, 158 – 170	-	-	-
	TRPV6	707	NP_001001849.1	-	-	-	-
	Sodium/potassium-transporting ATPase subunit alpha-2	1017	NP_571758.1	-	-	-	-
**Intestine**	Glycogen	421	NP_571456.1	177 – 189	(NP_571456.1)	1IO9	95.59
						4IQ6	95.59
						4PTC	95.59
	Glutaminase A	591	NP_001038509.1	-	-	-	-
**Brain**	Dopamine beta hydroxylase	614	AAI63055.1	53 – 169	(AAI63055.1)	4ZEL	62.91
						1SDW	26.71
						1YJW	26.71
	Acetylcholineeasterase	634	AAI63898	212 – 227	(AAI63898)	6EUC	66.23
						1DX6	65.78
						1ACJ	65.67
	Gmma-Aminobutyric acid	500	NP_958906	-	-		-
	Isotocin	154	AAL50209.1	-	-		-
**Liver**	Catalase	526	NP_570987.2	64 – 80, 354-362	(NP_570987.2)	3J7B	80.42
						3NWL	80.23
						1F4J	78.50
	GST	219	NP_997841.1	-	-	-	-
	Glutathione synthetase	475	NP_001006104.1	-	-	-	-
	IGF-1	161	NP_571900.1	-	-	-	-
	IP3	1447	Q5RHB5	-	-	-	-

Table S4. Tabular representation of best generated protein models showing DOPE score value and Ramachandran plot statistics after completing protein modelling of various organs of *Danio rerio.*

		**Ramachandran Plot Statistics (Only for best model based on the DOPE score)**			
Models	DOPE Score	Favourable region	Additionally allowed region	Generously allowed region	Unallowed region
**Intestine (Glycogen)**					
**Model1**	**-41623.52344**	**314 (86.3%)**	**45 (12.4%)**	**1 (0.3%)**	**4 (1.1%)**
Model 2	-41312.70703	-	-	-	-
Model 3	-41515.25781	-	-	-	-
**Brain (Dopamine)**					
Model 1	-46022.45703	-	-	-	-
**Model2**	**-46333.44531**	**396 (73.9%)**	**93 (17.4%)**	**30 (5.6%)**	**17 (3.2%)**
Model 3	-46082.64453	-	-	-	-
**(Acetylcholineeasterase)**					
**Model1**	**-72319.20313**	**489 (89.7%)**	**49 (9%)**	**6(1.1%)**	**1(0.2%)**
Model 2	-71752.47656	-	-	-	-
Model 3	-72291.82813	-	-	-	-
**Liver (Catalase)**					
**Model1**	**-52376.73438**	**415 (91.6%)**	**35 (7.7%)**	**2 (0.4%)**	**1 (0.2%)**
Model 2	-51820.67578	-	-	-	-
Model 3	-51958.68750	-	-	-	-

Table S5. Tabular representation of major proteins involved in calcium metabolism, signalling, and NRF-2 along with their functions in *Danio rerio.*

Organs	Proteins	Functions
**Gills**	Polo like kinase 1	Regulates cell cycle progression, mitotic spindle formation, and DNA damage repair, critical for cellular division and organ function.
	TRPV6	Mediates calcium uptake across epithelial tissues, essential for maintaining ionic balance and skeletal health.
	N-K ATPase alpha-2	Maintains sodium and potassium ion gradients, critical for osmoregulation and efficient gas exchange.
**Intestine**	Glutaminase A	Converts glutamine to glutamate, playing a role in nutrient metabolism and energy production.
	Glycogen	Serves as an energy reserve, critical for intestinal cellular functions and overall metabolism.
	Isotocin	Analogous to oxytocin, influences intestinal motility and fluid absorption, supporting digestive health.
**Brain**	Dopamine beta hydroxylase	Functions as a neurotransmitter regulating mood, cognition, and motor activity, potentially affected by oxidative stress.
	Acetylcholineeasterase	Hydrolyzes acetylcholine, facilitating neural signal termination and maintaining neurotransmission integrity.
	Glutathione Synthetase	Catalyzes the detoxification of harmful compounds, protecting neural tissues from oxidative damage.
**Liver**	Glutathione synthetase	Catalyzes glutathione biosynthesis, crucial for detoxifying reactive oxygen species and maintaining hepatic cellular health.
	IGF-1	Promotes calcium uptake and homeostasis by enhancing calcium-dependent signalling pathways essential for cellular growth and metabolic regulation.
	Catalase	Breaks down hydrogen peroxide into water and oxygen, protecting the liver from oxidative stress induced by CaO-NPs.
		**NRF2 pathway-related major proteins**
**Nuclear Factor Erythroid 2-Related Factor 2a (nfe2l2a)**		Regulates antioxidant and cytoprotective genes, indirectly influencing calcium signalling by modulating oxidative stress levels.
**Kelch-Like ECH-Associated Protein 1a(keap1a)**		Acts as an NRF2 inhibitor, modulating redox homeostasis and preventing excessive oxidative stress that affects calcium ion channels.
**Kelch-Like ECH-Associated Protein 1b (keap1b)**		Functions similarly to keap1a in regulating NRF2 activity, impacting calcium balance by maintaining redox equilibrium.

**Cullin 3a (cul3a)**	A component of the E3 ubiquitin ligase complex targeting NRF2, indirectly influencing calcium channel stability via redox balance.
**Nuclear Factor Erythroid 2-Related Factor 2b (nfe2l2b)**	Another NRF2 homologue involved in redox regulation, which can affect calcium-dependent signalling pathways.
**Nuclear Factor Erythroid 2-Related Factor 3 (nfe2l3)**	A transcription factor regulating antioxidant responses, influencing calcium signalling by reducing oxidative stress.
**Heme Oxygenase 1a (hmox1a)**	Encodes heme oxygenase-1, which reduces oxidative stress and supports calcium signalling through cellular homeostasis.
**Small Maf Protein K (mafK)**	A small Maf protein partnering with NRF2 to regulate antioxidant genes, indirectly modulating calcium signalling by reducing oxidative stress.
**Cullin 3b (cul3b)**	Another component of the E3 ubiquitin ligase complex, ensuring proper NRF2 turnover and redox stability, influencing calcium transport proteins.
**NAD(P)H Quinone Dehydrogenase 1 (nqo1)**	Reduces oxidative stress by detoxifying quinones, supporting calcium channel function indirectly.
